# Structural basis for abscisic acid efflux mediated by ABCG25 in *Arabidopsis thaliana*

**DOI:** 10.1038/s41477-023-01510-0

**Published:** 2023-09-04

**Authors:** Wei Ying, Lianghuan Liao, Hong Wei, Yongxiang Gao, Xin Liu, Linfeng Sun

**Affiliations:** 1 The First Affiliated Hospital of USTC, MOE Key Laboratory for Membraneless Organelles and Cellular Dynamics, Hefei National Research Center for Interdisciplinary Sciences at the Microscale, Division of Life Sciences and Medicine, University of Science and Technology of China, Hefei, China; 2 Biomedical Sciences and Health Laboratory of Anhui Province, University of Science and Technology of China, Hefei, China

**Keywords:** Plant hormones, Cryoelectron microscopy

## Abstract

Abscisic acid (ABA) is a phytohormone essential to the regulation of numerous aspects of plant growth and development. The cellular level of ABA is critical to its signalling and is determined by its rate of biosynthesis, catabolism and the rates of ABA transport. ABCG25 in *Arabidopsis thaliana* has been identified to be an ABA exporter and play roles in regulating stomatal closure and seed germination. However, its ABA transport mechanism remains unknown. Here we report the structures of ABCG25 under different states using cryo-electron microscopy single particle analysis: the apo state and ABA-bound state of the wild-type ABCG25 and the ATP-bound state of the ATPase catalytic mutant. ABCG25 forms a homodimer. ABA binds to a cone-shaped, cytosolic-facing cavity formed in the middle of the transmembrane domains. Key residues in ABA binding are identified and verified by a cell-based ABA transport assay. ATP binding leads to closing of the nucleotide-binding domains of opposing monomers and conformational transitions of the transmembrane domains. Together, these results provide insights into the substrate recognition and transport mechanisms of ABCG25 in *Arabidopsis*, and facilitate our understanding of the ABA transport and signalling pathway in plants.

## Abstract (web summary)

Cryo-EM structures are determined for the abscisic acid (ABA) exporter ABCG25 in *Arabidopsis* in the apo, ABA-bound and ATP-bound states, which facilitate our understanding of the ABA transport and signalling processes in plants.

## Main

The phytohormone abscisic acid (ABA) plays vital roles in a plethora of growth and development processes in plants, such as seed germination and dormancy, root growth, stomatal closure and leaf senescence^1–3^. It is also a key hormone regulating plant adaptations to a variety of abiotic or biotic stresses, such as drought, salinity, cold or heat and pathogen invasions^4–6^. Since its first discovery in 1960s, the ABA perception and signalling pathway has been deeply characterized, with the identification of multiple ABA receptors, regulators and their interaction network, including the Pyracbactin Resistance/Pyracbactin resistance-like/Regulatory Component of ABA Receptor proteins as the key ABA receptors, and Protein Phosphatase 2Cs and SNF1-related protein kinase 2s as the major negative regulators and positive regulators, respectively^7–15^. Local ABA concentrations are critical to initiating the signalling cascade and its functions, and are tightly regulated by its biosynthesis, catabolism and transport pathways^16,17^. Lines of evidence have proved the existence of ABA transport both at cellular/local and long-distance levels in several plant tissues and organs, including compartmental distribution and redistribution in root and leaf, root-to-shoot movement via the xylem in regulating stomatal closure, shoot-to-root transport in promoting root growth and leaf-to-seed transport to facilitate normal seed development^17–24^.

**Extended Data Fig. 1:**
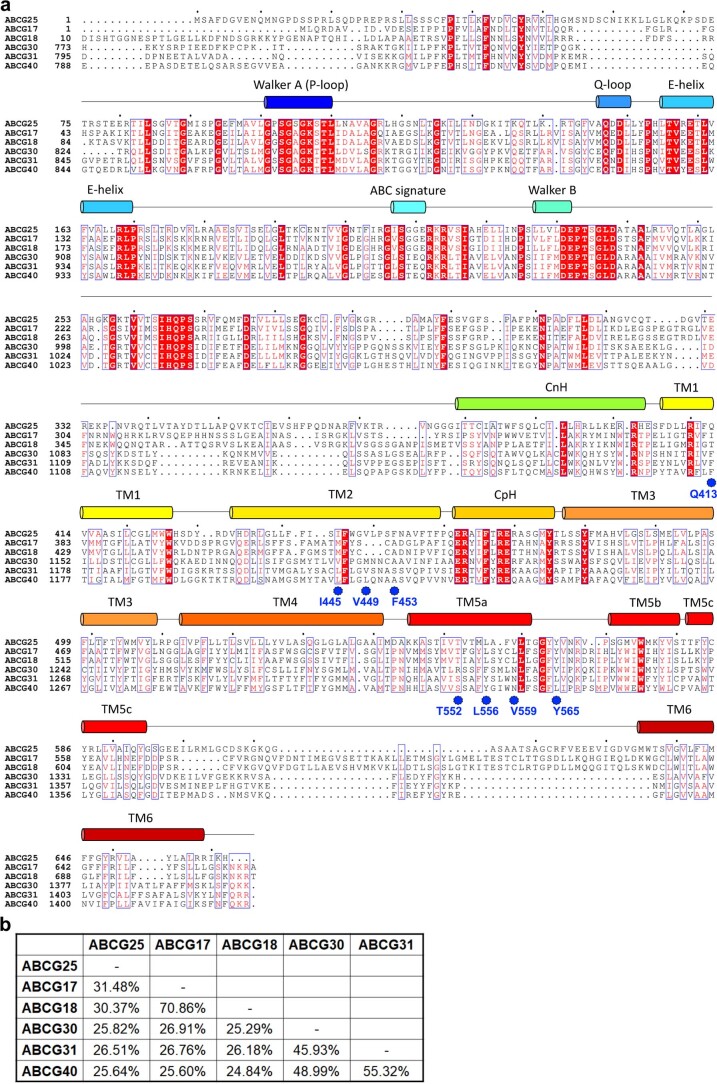
Sequence alignments of ABCG family ABA transporters in *Arabidopsis thaliana*. **a**, Sequence alignments of the six ABCGs as ABA transporters in *Arabidopsis thaliana*. The amino acid sequences are available at Uniprot (https://www.uniprot.org) with the following accession codes: ABCG25 - Q84TH5, ABCG17 - Q9M2V6, ABCG18 - Q9M2V5, ABCG30 - Q8GZ52, ABCG31 - Q7PC88, ABCG40 - Q9M9E1. Sequences are aligned using Clustal O (1.2.4). Secondary structural elements of ABCG25 are indicated above the sequence. Invariant amino acids are shaded red. The ABA interacting residues are indicated by blue stars. **b**, Amino acid sequence identities between the ABCG family ABA transporters in *Arabidopsis thaliana*.

**Extended Data Fig. 2:**
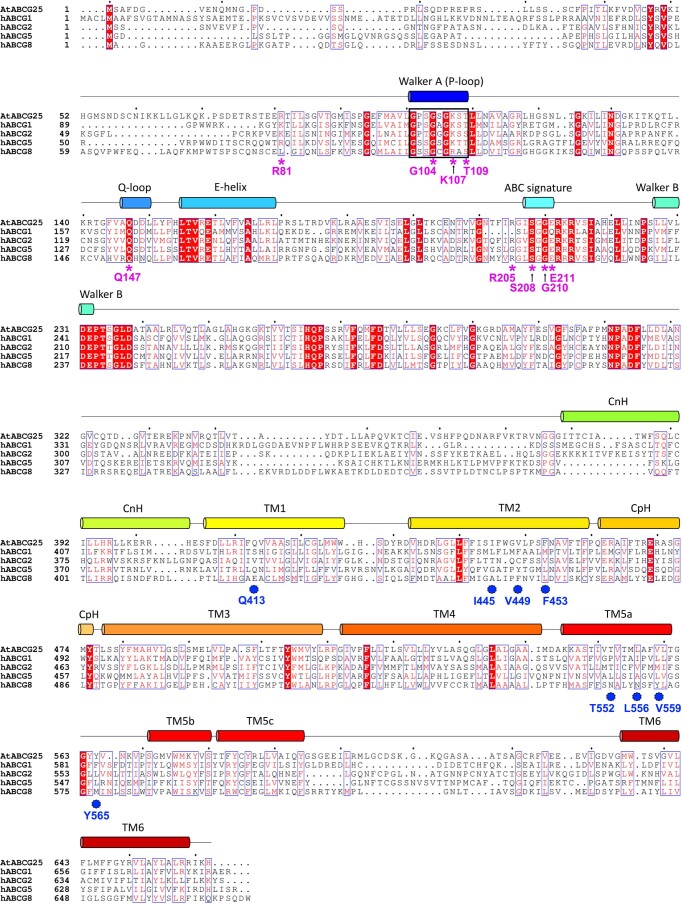
Sequence alignments of ABCG25 with human ABCGs. Sequence alignments of ABCG25 and its human homologues with determined structures, including hABCG1, hABCG2, hABCG5 and hABCG8. The amino acid sequences are available at Uniprot (https://www.uniprot.org) with the following accession codes: ABCG25 - Q84TH5, hABCG1 - P45844, hABCG2 - Q9UNQ0, hABCG5 - Q9H222, hABCG8 - Q9H221. Sequences are aligned using Clustal O (1.2.4). Secondary structural elements of ABCG25 are indicated above the sequence. Invariant amino acids are shaded red. Key residues involved in ATP-binding and hydrolysis are indicated by pink star. Residues surrounding ABA are indicated by blue stars.

As a 15-carbon, sesquiterpene-type weak acid with a p*K*_a_ (the negative base 10 logarithm of the acid dissociation constant) value of ~4.7, ABA is partially in the protonated uncharged form (ABAH) in the acidic apoplast environment and can diffuse freely across the plasma membrane, a passive influx process similar to that in another phytohormone, auxin^1^. Meanwhile, transporter-mediated active ABA influx has also been identified, which may overcome the limited rate and efficiency of passive diffusion^17,25^. Such importers include members of the ATP-binding cassette (ABC) subfamily G, such as ABCG40 which imports ABA into guard cells to regulate stomatal closure, ABCG30 which imports ABA from the endosperm into the embryo to control seed germination, ABCG17 and ABCG18 which are primarily expressed in the shoot mesophyll and stem cortex cells and regulate stomatal closure as well as lateral root emergence^26–28^. In addition, the ABA-importing transporters 1–4 (also known as NPF4.6, 4.5, 4.1 and 4.2, respectively) and the PLASMA MEMBRANE PROTEIN 1 in rice (*Os*PM1) have also been identified to be ABA importers^29–32^. Once inside of the cell, the protonated ABA dissociates into the anion form (ABA^−^) due to the elevation in cytosolic pH, and its movement is restricted. Thus, transporters are required for the ABA efflux process. The Detoxification Efflux Carriers (DTX)/Multidrug and Toxic Compound Extrusion (MATE) family transporter DTX50 in *Arabidopsis* mainly expressed in vascular tissues was found to mediate ABA efflux and drought stress responses^33^. Another MATE transporter, DG1 in rice, was also shown to be an ABA exporter that regulates the leaf-to-caryopsis ABA long-distance transport to control seed development^23^. Several ABCG family transporters also function as ABA exporters. ABCG20 in *Medicago truncatula* was identified as an ABA exporter in roots and germinating seeds, and influenced root morphology and seed germination^34^. ABCG25 in *Arabidopsis* was the first ABA exporter identified through genetic screening for ABA-sensitive phenotypes^35^. It is mainly expressed in vascular tissues, as well as in the endosperm where it functions together with another ABA exporter, ABCG31, to export ABA from the endosperm to the embryo and regulate seed germination^26^. The *atabcg25* knockout plants show no altered aerial or germination phenotypes under normal growth conditions^26,35,36^, possibly due to the existence of redundant members, but are hypersensitive to ABA treatment^35^, and the seeds exhibit retarded embryonic growth^26^. Overexpression of ABCG25 leads to a higher leaf temperature and slower rate of water loss, suggesting a role in stomatal regulation^35^. Although several ABCG proteins have been identified as ABA transporters, the variations in transport directions, expression patterns and phenotypes of mutants suggest non-redundant roles in plants. In addition, protein sequences among such ABCGs vary largely (Extended Data Fig. 1a,b). ABCG25 in *Arabidopsis* share only ~30% sequence identity with ABCG17 or ABCG18 and less than 27% with ABCG30, ABCG31 or ABCG40 (Extended Data Fig. 1a,b). The molecular mechanism of their ABA recognition remains unknown. The protein sequence of ABCG25 in *Arabidopsis* also varies from that of human ABCGs with determined structures (<30% sequence identities; Extended Data Fig. 2), especially in the transmembrane region.

In this study, we determined the high-resolution structures of ABCG25 in *Arabidopsis thaliana* in the apo, ABA-bound and ATP-bound states using cryogenic electron microscopy (cryo-EM) single particle analysis. The structural studies, together with the biochemical analysis, depict the architecture of ABCG25, how it recognizes ABA and the structural transitions during ABA transport.

## Results

### ABCG25 utilizes ATP hydrolysis to drive ABA efflux

**Fig. 1:**
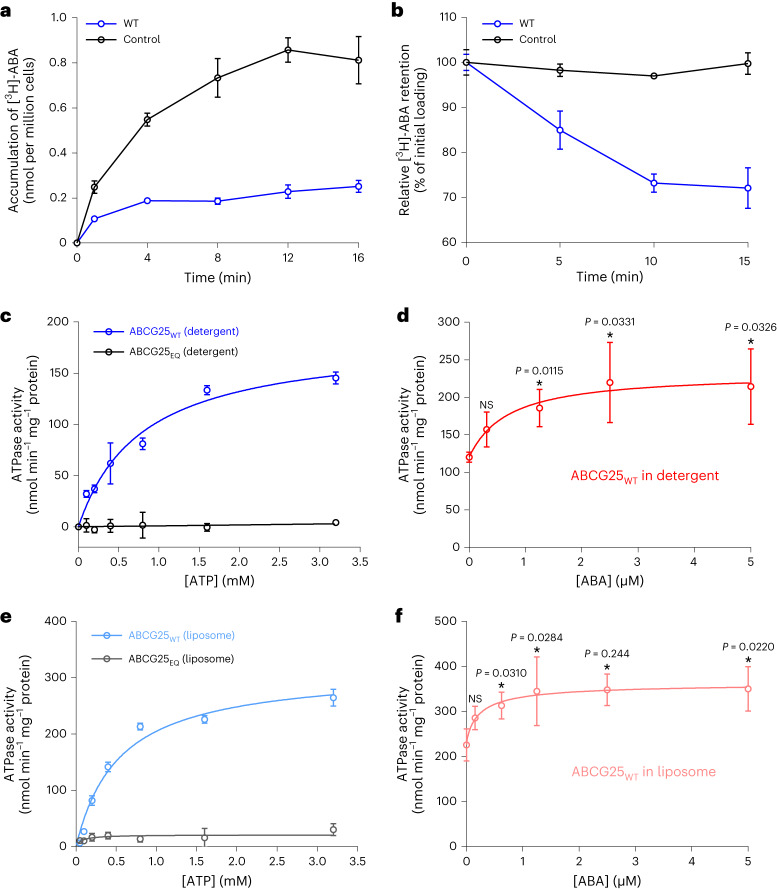
Characterization of ABCG25-mediated ABA transport and ATPase activity. **a**, Loading assay showing that accumulation of [^3^H]-ABA is decreased in cells expressing ABCG25 compared with untransfected control cells. All data points represent five independent measurements. Data are mean ± s.d. **b**, Efflux assay showing that [^3^H]-ABA retention is decreased in cells expressing ABCG25 compared with control cells. Data points represent three independent measurements. Data are mean ± s.d. **c**, ATPase activities of ABCG25_WT_ and ABCG25_EQ_ in detergent micelle in the presence of different concentrations of ATP. Data points were nonlinear-fitted using the Michaelis–Menten equation. Data points represent four independent measurements. Data are mean ± s.d. **d**, ABA concentration-dependent ATPase activity of ABCG25_WT_ in detergent micelle. Data points were nonlinear-fitted using the allosteric sigmoidal model. Data points represent three independent measurements. NS, not significant; *P < 0.05 for ABA-added versus no ABA (one-way analysis of variance (ANOVA) with Dunnett’s multiple comparisons test). Data are mean ± s.d. **e**, ATPase activities of ABCG25_WT_ and ABCG25_EQ_ reconstituted into liposomes in the presence of different concentrations of ATP. Data points were nonlinear-fitted using the Michaelis–Menten equation. Data points represent four independent measurements. Data are mean ± s.d. **f**, ABA concentration-dependent ATPase activity of ABCG25_WT_ reconstituted into liposomes. Data points were nonlinear-fitted using the allosteric sigmoidal model. Data points represent three independent measurements. *P < 0.05 for ABA-added versus no ABA (one-way ANOVA with Dunnett’s multiple comparisons test). Data are mean ± s.d. Source data

**Extended Data Fig. 3:**
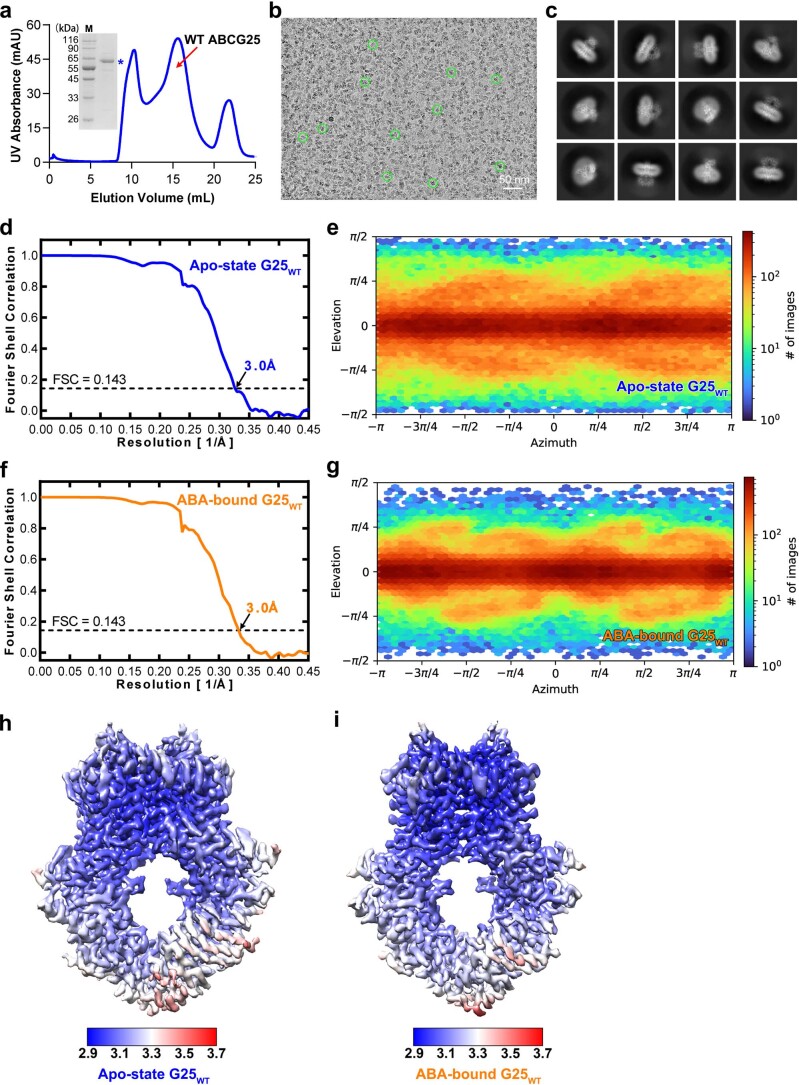
Cryo-EM analysis of ABCG25_WT_ in the apo- or ABA-bound state. **a**, A representative gel filtration and Coomassie-blue-staining SDS-PAGE result of wild-type ABCG25 purified with DDM plus CHS extraction. Experiments have been repeated for at least three times with similar results. Uncropped gel can be found in Source Data. **b**, A typical cryo-EM image of the apo-state ABCG25_WT_ (DDM plus CHS extraction). Experiments have been repeated for at least three times with similar results. **c**, Typical 2D classification images of the apo-state ABCG25_WT_ (DDM plus CHS extraction). Experiments have been repeated for at least three times with similar results. **d**, The gold-standard Fourier shell correlation curve for the overall map of the apo-state ABCG25_WT_ (DDM plus CHS extraction). The resolution reaches 3.0 Å by a threshold of 0.143. **e**, Euler angle distribution of the apo-state ABCG25_WT_ (DDM plus CHS extraction). **f**, The gold-standard Fourier shell correlation curve for the overall map of the ABA-bound ABCG25_WT_ (DDM plus CHS extraction). The resolution reaches 3.0 Å by a threshold of 0.143. **g**, Euler angle distribution of the ABA-bound ABCG25_WT_ (DDM plus CHS extraction). **h**, The local resolution map of the apo-state ABCG25_WT_ (DDM plus CHS extraction) calculated using ResMap. **i**, The local resolution map of the ABA-bound ABCG25_WT_ (DDM plus CHS extraction) calculated using ResMap. Source data

The ABA efflux activity of ABCG25 from *Arabidopsis thaliana* has been established in heterologous systems, including using *Xenopus* oocytes, *Spodoptera frugiperda* 9 (Sf9) insect cells, or membrane vesicles from ABCG25-expressing Sf9 insect cells^35,37,38^. We tried to set up an Sf9 cell-based assay system using radioisotope-labelled ABA ([^3^H]-ABA) to evaluate its transport activity. By employing the chemical property that ABA can diffuse freely across membranes under an acidic pH environment, two assays were derived: a loading assay which measures the radioisotope-labelled ABA accumulation in the cell and an efflux assay which measures residual radiolabeled ABA over time after transferring the cells into the isotope-free buffer. Successful expression and good behaviour of ABCG25 in Sf9 cells were verified by protein purification and gel filtration (Extended Data Fig. 3a). In the loading assay, cells expressing ABCG25 accumulated much less [^3^H]-ABA at the indicated time compared with untransfected control cells (Fig. 1a). In the efflux assay, residual [^3^H]-ABA substantially decreased in cells expressing ABCG25, whereas it remained almost unchanged in control cells over the incubation period (Fig. 1b). Together, these results further confirmed that ABCG25 is able to mediate ABA efflux in Sf9 cells.

With the purified ABCG25 protein in detergent micelle, we first tested its ATPase activity in the presence of different concentrations of ATP. For the wild-type (WT) ABCG25 (ABCG25_WT_), the fitted maximum rate (*V*_max_) of ATP hydrolysis was 184.4 nmol^−1^ ATP min^−1^ mg^−1^ ABCG25 protein, with a Michaelis–Menten constant (*K*_m_) of 0.79 mM (Fig. 1c). We generated a catalytic mutant by replacing the glutamate residue E232 in the conserved Walker B motif of the nucleotide-binding domain (NBD) with a glutamine (named ABCG25_EQ_ hereafter). Such a mutant totally lost its ATP hydrolysis activity (Fig. 1c). We then tested the ATPase activity of ABCG25_WT_ under different ABA concentrations. The result showed a weak concentration-dependent stimulation of the ATPase activity of ABCG25 by ABA, with a half-maximal effective concentration value (EC_50_) of 0.68 μM (Fig. 1d). Notably, the basal activity of ABCG25 in the absence of ABA was also relatively high, at ~120.0 nmol^−1^ ATP min^−1^ mg^−1^ protein (Fig. 1d). We also reconstituted the purified ABCG25 protein into liposomes and tested its ATPase activity. For the ABCG25_WT_, the fitted *V*_max_ of ATP hydrolysis in response to the ATP concentration was 315.1 nmol^−1^ ATP min^−1^ mg^−1^ protein, nearly twofold of that determined for protein in detergent micelle, with a Michaelis–Menten constant (*K*_m_) of 0.53 mM similar to that determined in detergent micelle (Fig. 1e). We also observed weak increases in the ATPase activity of ABCG25 with increasing ABA concentrations (Fig. 1f).

### Architecture of ABCG25_WT_ in the absence of ABA

**Fig. 2:**
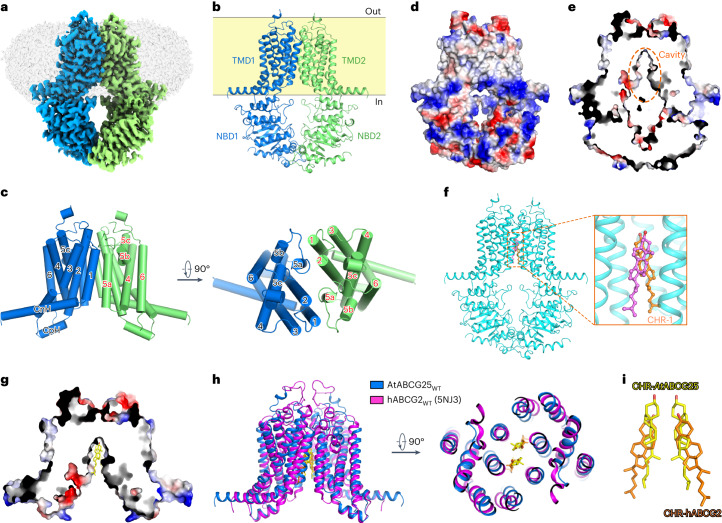
Architecture of ABCG25_WT_ in the apo state. **a**, Overview of the electron density for the ABCG25_WT_ (purified with DDM plus CHS extraction) in the apo state. Densities corresponding to the ABCG25 monomers are coloured blue and green. The detergent density is shown in grey. **b**, Overall structure of the apo-state ABCG25_WT_. **c**, A cartoon representation of the TMDs of the apo-state ABCG25_WT_. **d**, Overview of the surface electrostatic potential of the apo-state ABCG25_WT_ from the side of the membrane. Negative and positive charges are coloured red and blue, respectively. **e**, Section view of the surface electrostatic potential of the apo-state ABCG25_WT_. The cavity is indicated with orange dashed lines. **f**, Two cholesterol molecules are modelled in the intracellular pocket according to the observed densities in the EM map of ABCG25_WT_ (purified with DDM plus CHS extraction) in the apo state. **g**, Section view of the surface electrostatic potential of ABCG25_WT_ in the apo state, with the cholesterol molecules shown as sticks. **h**, Structural alignment of ABCG25 and hABCG2 (PDB code: 5NJ3) in the cholesterol-bound state. ABCG25 and hABCG2 are coloured blue and magenta, respectively. Carbon atoms of the cholesterol molecules in ABCG25 and hABCG2 are coloured yellow and orange, respectively. **i**, Superposition of the cholesterol molecules observed in ABCG25 (yellow) and hABCG2 (orange, PDB code: 5NJ3).

**Extended Data Fig. 4:**
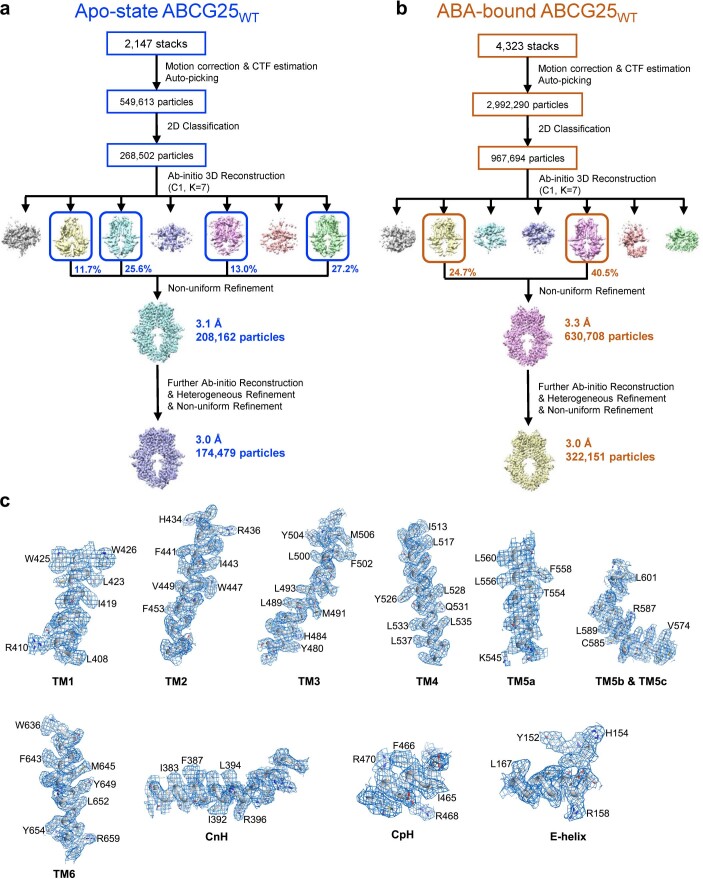
Flowcharts for cryo-EM data processing of ABCG25_WT_ in the apo- or ABA-bound state. **a**, A flowchart for the data processing of ABCG25_WT_ with DDM plus CHS extraction in the apo-state. Details can be found in the ‘Image processing’ session in Methods. **b**, A flowchart for the data processing of ABCG25_WT_ (DDM plus CHS extraction) in the ABA-bound state. Details can be found in the ‘Image processing’ session in Methods. **c**, EM densities for the six transmembrane helices, CnH, CpH and E-helix of the apo-state ABCG25_WT_ (DDM plus CHS extraction) map. Residues with notable side chains are labeled aside.

**Extended Data Fig. 5:**
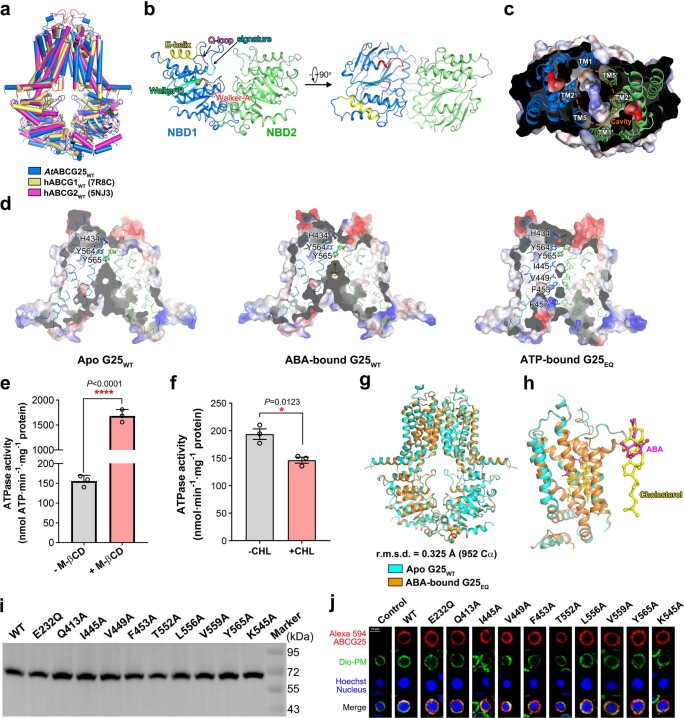
Structure analysis of ABCG25. **a**, Structure alignments of ABCG25_WT_ and hABCG1_WT_ (PDB code: 7R8C), hABCG2WT (PDB code: 5NJ3) in the apo-state. **b**, The separated NBDs of ABCG25_WT_ in the apo-state. The conserved motifs such as walker A, Q-loop, ABC signature, walker B, and E-helix are indicated. **c**, The substrate-binding cavity formed by TM1, TM2 and TM5a from the opposing monomers. **d**, Section view of the surface electrostatic potential of ABCG25 TMDs in the apo-, ABA-bound or ATP-bound state. The residues at the exit of transport cavity and along the substrate translocation pathway are labeled aside and shown as sticks. **e**, ATPase activities of ABCG25_WT_ in detergent micelle in the absence or presence of 0.5mM M-βCD. Three independent experiments were performed for each construct (n = 3). **** *P* < 0.0001 (two-tailed unpaired *t*-test). Data are mean ± SEM. **f**, ATPase activities of ABCG25_WT_ in liposome reconstituted with or without cholesterol (lipid:cholesterol prepared at a 9:1(w/w) ratio). Three independent experiments were performed for each construct (n = 3). * *P* = 0.0123 (two-tailed unpaired *t*-test). Data are mean ± SEM. **g**, Structure alignments of ABCG25_WT_ in the apo- and ABA-bound state. The ABA-bound structure (coloured orange) is almost identical to the apo-state structure (coloured cyan). **h**, Zoom-in views of the substrate-binding site of ABCG25 in the apo- and ABA-bound state and superposition of ABA and cholesterol. **i**, Western blotting analysis of the expression level of ABCG25 variants using anti-Flag antibody. Experiments have been repeated at least twice with similar results. Uncropped blot can be found in Source Data. **j**, Confocal microscopy representative images illustrating the localization of ABCG25 variants. Empty or ABCG25-expressing Sf9 cells were immunostained with anti-Flag Alexa Fluor 594-conjugated antibody and counterstained with Dio and Hoechst 33342. All the variants have an N-terminal Flag tag. Red, Alexa 594, ABCG25; green, Dio, plasma membrane; blue, Hoechst 33342, cell nucleus; merge, overlay among Alexa 594, Dio and Hoechst 33342. Scale bar = 10 μm. Experiments have been repeated at least twice with similar results. Source data

The ABCG25_WT_ protein purified using dodecyl maltopyranoside (DDM) plus cholesteryl hemisuccinate (CHS) extraction behaved well on gel filtration, suitable for cryo-EM sample preparation (Extended Data Fig. 3a). After data collection and processing, an EM map with an overall resolution of 3.0 Å as estimated by the gold-standard Fourier shell correlation method was finally obtained, allowing de novo model building (Fig. 2a, Extended Data Figs. 3b–e,h and 4a,c and Table 1). As a ‘half-transporter’ with only one NBD and one transmembrane domain (TMD) in a polypeptide chain, ABCG25 forms a homodimer as a functional unit, similar to other determined ‘half-transporter’ ABCG structures including the human cholesterol transporter ABCG1 (hABCG1) and multidrug transporter ABCG2 (hABCG2)^39–47^ (Fig. 2a,b). Structural alignments with the WT structure of human ABCG1 or ABCG2 in the apo state result in a root-mean-square deviation (RMSD) of 3.60 and 3.53 Å, respectively, with a similar fold in the TMD or NBD (Extended Data Fig. 5a). In ABCG25, the NBDs of opposing monomers are mostly separated, especially for the putative ATP-binding sites formed by the Walker A motif (or P-loop), Q-loop and Walker B motif from one NBD, and the signature sequence from the other (Extended Data Fig. 5b). The TMD of each monomer consists of six transmembrane segments (TMs), TM1–TM6. The extracellular part of TM5 breaks into two short helices, TM5b and TM5c, with TM5a largely tilted towards the membrane plane (Fig. 2c). Part of the extracellular loop between TM5c and TM6 was unresolved due to vague EM density. A long helix before TM1, which lies almost parallel to the membrane plane, forms the connecting helix (CnH), and a short helix between TM2 and TM3 forms the coupling helix (CpH) (Fig. 2c). These two helices, together with a helix from the NBD (E-helix, containing a conserved glutamic acid residue, E159 in ABCG25) constitute a signature three-helix bundle that is observed in all determined structures of ABCGs.

**Extended Data Fig. 6:**
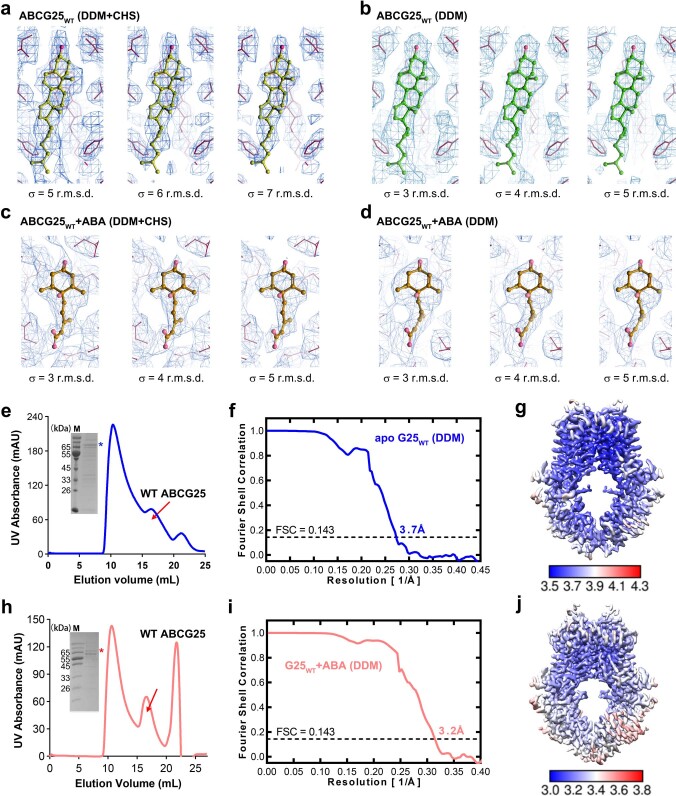
EM densities for the cholesterol and ABA molecules in ABCG25_WT_. **a**, The EM densities interpreted as cholesterol molecules in the apo-state ABCG25_WT_ (DDM plus CHS extraction) map. The maps are prepared in three different contour levels using Coot. **b**, The EM densities interpreted as cholesterol molecules in the apo-state ABCG25_WT_ (DDM alone extraction) map. The maps are prepared in three different contour levels using Coot. **c**, The EM densities of ABA molecules in the ABA-bound ABCG25_WT_ (DDM plus CHS extraction) map. The maps are prepared in three different contour levels using Coot. **d**, The EM densities of ABA molecules in the ABA-bound ABCG25_WT_ (DDM extraction with ABA added) map. The maps are prepared in three different contour levels using Coot. **e**, A representative gel filtration and Coomassie-blue-staining SDS-PAGE result of wild-type ABCG25 purified with DDM alone extraction. Experiments have been repeated for at least three times with similar results. Uncropped gel can be found in Source Data. **f**, The gold-standard Fourier shell correlation curve for the overall map of the apo-state ABCG25_WT_ (DDM extraction). The resolution reaches 3.7 Å by a threshold of 0.143. **g**, The local resolution map of the apo-state ABCG25_WT_ (DDM extraction) calculated using ResMap. **h**, A representative gel filtration and Coomassie-blue-staining SDS-PAGE result of wild-type ABCG25 purified with DDM extraction and ABA-added during the whole purification steps. Experiments have been repeated for at least three times with similar results. Uncropped gel can be found in Source Data. **i**, The gold-standard Fourier shell correlation curve for the overall map of the ABA-bound ABCG25_WT_ (DDM extraction with ABA added). The resolution reaches 3.2 Å by a threshold of 0.143. **j**, The local resolution map of the ABA-bound ABCG25_WT_ (DDM extraction with ABA added) calculated using ResMap. Source data

A section view of the electrostatic potential surface reveals an inward-facing cavity in the transmembrane region of ABCG25 (Fig. 2d,e). The cavity, which is mainly neutral but has a weak positive electrostatic potential at the bottom, is formed by TM1, TM2 and TM5a from the opposing monomers (Extended Data Fig. 5c). The cavity is sealed from the extracellular space by three layers of bulky residues from opposing monomers stacking together, including H434 on TM2, Y564 and Y565 on TM5a (Extended Data Fig. 5d). Y565 also contributes to the weak positive electrostatic potential at the bottom of the cavity. Although no ABA substrate was added during protein purification and cryo-sample preparation, two clear, long-tailed densities were observed in this cavity (Fig. 2f and Extended Data Fig. 6a). Since CHS was added during protein purification, we first guessed that these might be the CHS densities. However, the density is too small to accommodate the whole CHS molecule and does not fit well. We also tried to remove CHS during protein purification and extracted the protein from the membrane using DDM alone. The removal of CHS led to dramatically decreased protein yield, indicating a critical role of CHS in stabilizing the protein (Extended Data Fig. 6e). Nonetheless, with the purified protein, we obtained an EM map with an overall resolution of 3.7 Å (Extended Data Fig. 6f,g). The protein structure remained almost unchanged compared to that determined with DDM plus CHS extraction, with an RMSD of 0.33 Å. However, in this map, we could still observe the two identical densities in the cavity (Extended Data Fig. 6b), further supporting the idea that they are not contributed by CHS. We speculated that they may be some endogenous sterols in Sf9 cells. We used cholesterol as a representative of the endogenous sterols, although the cholesterol content in Sf9 cells is minimal^48^, and tried to dock it into the EM densities. The two densities can be docked well with two cholesterol molecules, with the hydrophilic head pointing towards the bottom of the cavity (Fig. 2f,g). When methyl-β-cyclodextrin (M-βCD), which can selectively bind cholesterol^49^, was added to the protein, its ATPase activity was dramatically increased by ~10-fold (Extended Data Fig. 5e). Reciprocally, when cholesterol was added during liposomes reconstitution, the ATPase activity of ABCG25 was significantly reduced (Extended Data Fig. 5f), suggesting that the observed densities might be contributed by sterols. However, we noted that the addition of a high concentration M-βCD also led to destabilization of the protein. Thus, the observed increase in ATPase activity due to M-βCD addition could also be caused by other changes in the protein, and the role of sterols in ABCG25 function remains to be further investigated. In addition, since the identities of the densities were not entirely determined, they can also be other endogenous molecules in the Sf9 cells. Interestingly, in the structure of hABCG2 determined with an antibody fragment, cholesterol molecules, which are not its physiological substrates, were also found in the cytosolic-facing cavity. Structure alignments reveal that the cholesterol molecules bind to a similar site in ABCG25 or hABCG2 (Fig. 2h,i), indicating a possibly conserved regulatory role of sterols in the transport activity.

### ABA binding site in ABCG25

**Fig. 3:**
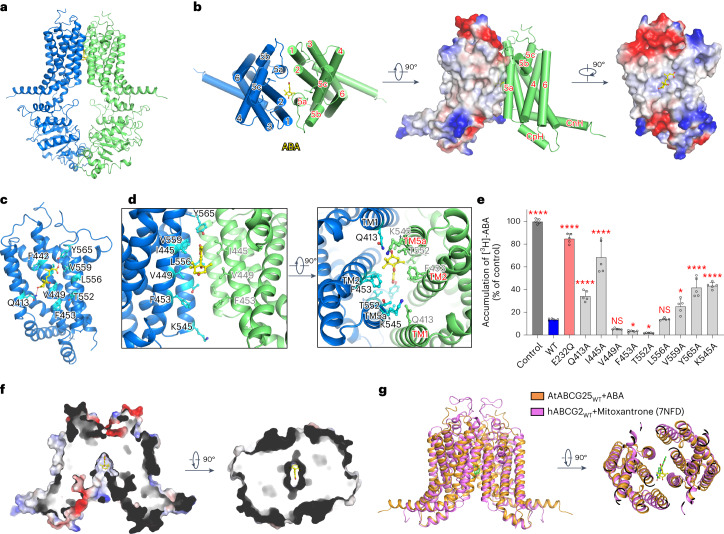
Architecture of ABCG25_WT_ in the ABA-bound state. **a**, Overall structure of ABCG25_WT_ (purified with DDM plus CHS extraction) in the ABA-bound state. ABA is shown in sticks. EM density is shown for ABA using Chimera. **b**, A cartoon representation of the TMDs of the ABA-bound ABCG25_WT_ and electrostatic surface representation of the ABA binding site. ABA binds to a hydrophobic pocket of ABCG25 in the transmembrane region. **c**, Coordination of ABA by the ABCG25 monomer. Side chains of the residues close to ABA are shown in sticks, with carbon atoms coloured cyan. **d**, Zoom-in views of the ABA molecule and the surrounding residues. TMD1 is shown in blue and TMD2 is shown in green. **e**, Characterization of ABA transport for WT and mutant ABCG25 in Sf9 cells using the ABA loading assay. Five independent experiments were performed for each construct. NS, not significant; *P = 0.0439 for F453A, 0.0138 for T552A and 0.0151 for V559A; ****P < 0.0001 for other mutants versus WT (one-way ANOVA with Dunnett’s multiple comparisons test). Data are mean ± s.d. **f**, Section view of the surface electrostatic potential of the ABA-bound ABCG25_WT_, with ABA shown as sticks. **g**, Structural alignment of ABCG25 and hABCG2 (PDB code: 7NFD) in the substrate-bound state. ABCG25 and hABCG2 are coloured orange and pink, respectively. ABA (yellow) and mitoxantrone (green) are shown as sticks. Source data

To reveal the substrate recognition mechanism of ABCG25, we tried to obtain a complex structure by adding ABA to the purified WT protein with DDM plus CHS extraction and incubating before cryo-sample preparation. An EM map with an overall resolution of 3.0 Å was determined (Fig. 3a and Extended Data Figs. 3f,g,i and 4b). This structure is almost identical to the apo-state ABCG25 structure, as structure alignments reveal an RMSD of 0.33 Å (Extended Data Fig. 5g). It is also in an inward-facing state, with a cone-shaped cavity formed by the opposing TMDs (Fig. 3f and Extended Data Fig. 5d). In the cavity, clear densities were observed. Due to steric clashes in the carboxylate group, one ABA molecule can be fitted well into the density (Fig. 3a and Extended Data Fig. 6c). Since ABCG25 exhibits a twofold symmetry, ABA can also bind in the opposite orientation. We also purified ABCG25_WT_ protein with DDM extraction and ABA addition during the whole purification step (Extended Data Fig. 6h), and finally determined an EM map with an overall resolution of 3.2 Å (Extended Data Fig. 6i,j). The protein structure is almost identical to that determined with DDM plus CHS extraction, with an RMSD of 0.25 Å. A clear density was also observed in the identical site in protein sample prepared with DDM plus CHS, which can be docked by one ABA molecule with a better fitting density (Extended Data Fig. 6d), further supporting the ABA binding site. ABA lies almost at the middle of the TMDs, with the carboxylate tail pointing to the bottom of the cavity and the ring head group pointing to the cytoplasmic side (Fig. 3a,b,f). Notably, ABA merges well in the relative position, with the interpreted cholesterol molecules observed in the apo-state structure (Extended Data Fig. 5h).

The interface between ABA and ABCG25 is mainly formed by TM2 and TM5a (Fig. 3b). The surrounding residues are hydrophobic or polar in nature, including Q413 on TM1; F442, I445, V449 and F453 on TM2 of one subunit; and T552, L556 and V559 on TM5 of the other subunit (Fig. 3c,d). The phenolic hydroxyl groups of Y565 form a hydrogen bond with the carboxylate group of ABA, and Q413 interacts with the ketone group of the cyclohexene ring (Fig. 3d). In addition, as in the apo-state structure, Y565, Y564 and H434 seal the TMD to the extracellular space and form a gate for ABA release (Extended Data Fig. 5d). To evaluate the functions of the residues surrounding ABA, we generated a series of ABCG25 variants by mutating each of the residues to alanine and examined their ABA transport activities using the ABA loading assay. Western blotting and immunofluorescence staining of the mutants revealed that the mutations lead to no effects on protein expression or membrane localization (Extended Data Fig. 5i,j). As shown by the transport results, the ATPase loss-of-function mutant E232Q accumulated more [^3^H]-ABA than the WT, similar to the control group, suggesting a loss of ABA transport activity (Fig. 3e). For Q413A, I445A, V559A or Y565A, the accumulated [^3^H]-ABA was also significantly higher than that in the ABCG25_WT_, particularly for I445A, suggesting a severely impaired ABA efflux activity (Fig. 3e). For V449A, F453A or L556A, there was no significant difference compared to the WT protein (Fig. 3e), whereas for T552A, the [^3^H]-ABA accumulation was less than that in the WT protein, indicating enhanced ABA efflux activity (Fig. 3e). These results suggest that residues forming the bottom part of the cavity, including I445, V559 and Y565, are critical to ABA recognition and efflux, while single mutation of the residues along the ring group of ABA, including V449, F453 and L556, has little effect on ABA efflux.

**Extended Data Fig. 7:**
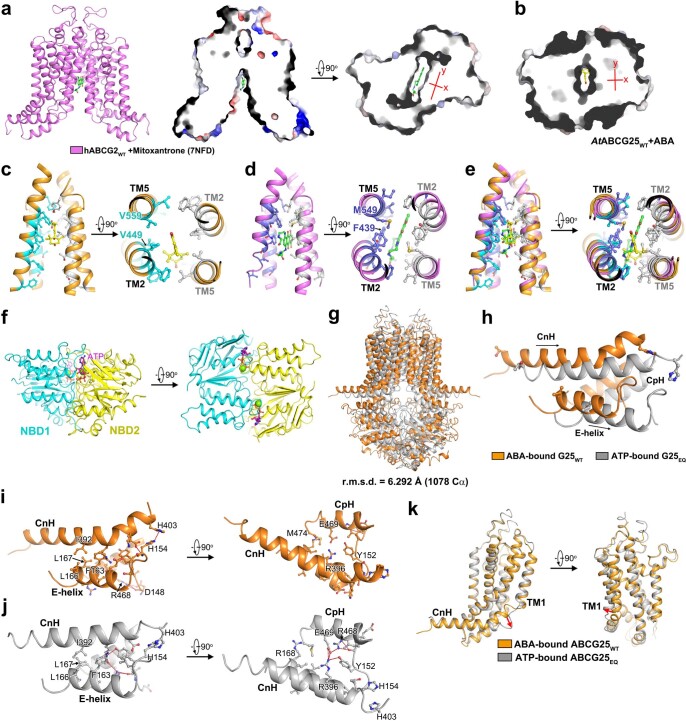
Comparison of the substrate-binding cavity between ABCG25 and ABCG2. **a**, The substrate-binding cavity of the mitoxantrone-bound hABCG2_WT_ (7NFD). Mitoxantrone (coloured green) is shown as sticks. The binding cavity exhibits a more planar and elongated feature, as shown by the surface electrostatic potential view. **b**, A view of the surface electrostatic potential of the substrate-binding cavity of the ABA-bound ABCG25_WT_. ABA (coloured yellow) is shown as sticks. **c**, Zoom-in views of residues in the substrate-binding cavity of the ABA-bound ABCG25_WT_. The ABA molecule and the surrounding residues are shown as sticks. **d**, Zoom-in views of residues in the substrate-binding cavity of the mitoxantrone-bound hABCG2_WT_ (7NFD). The mitoxantrone molecule and the interacting residues are shown as sticks. **e**, Structure alignments of the substrate-binding cavity between the ABA-bound ABCG25_WT_ and the mitoxantrone-bound hABCG2_WT_ (7NFD). **f**, The closed NBDs of ABCG25_EQ_ in the ATP-bound state. **g**, Structure alignments of ABCG25 in the ABA-bound (coloured orange) and ATP-bound (coloured gray) state. **h**, Superposition of the three-helix bundle of ABCG25 in the ABA-bound (coloured orange) and ATP-bound state (coloured gray). **i**, Zoom-in views of the three-helix bundle formed by CnH, CpH and E-helix in the ABA-bound ABCG25_WT_. The interacting residues are shown as sticks. Hydrogen bonds are shown in red dashed lines. **j**, Zoom-in views of the three-helix bundle formed by CnH, CpH and E-helix in the ATP-bound ABCG25_EQ_. The interacting residues are shown as sticks. Hydrogen bonds are shown in red dashed lines. **k**, Comparison of the TMDs during structure transitions between the ABA-bound (coloured orange) and ATP-bound (coloured gray) states of ABCG25. The rotation of the C-terminal part of CnH is indicated by the red arrows.

hABCG2 has been extensively studied, with complex structures determined for multiple substrates^40,44–46^. Similar to ABCG25, the substrates of hABCG2 are mostly hydrophilic and different substrates bind with a similar pattern^40,44–46^. We compare our ABA-bound structure with hABCG2 bound to a representative substrate, mitoxantrone^45^ (Extended Data Fig. 7a). Structure alignments reveal that ABA and mitoxantrone bind to similar positions in the cavity of each transporter, mainly formed by TM2 and TM5 (Fig. 3g and Extended Data Fig. 7c,d). However, since mitoxantrone has a planar anthraquinone structure, the binding cavity of hABCG2 also exhibits a more planar feature (Extended Data Fig. 7a). For ABCG25, residues surrounding the carboxylate group of ABA have smaller side chains, especially V449 (F439 in hABCG2) and V559 (M549 in hABCG2) (Extended Data Fig. 7b,c,e).

### Structure of the ABCG25 catalytic mutant in the ATP-bound state

**Fig. 4:**
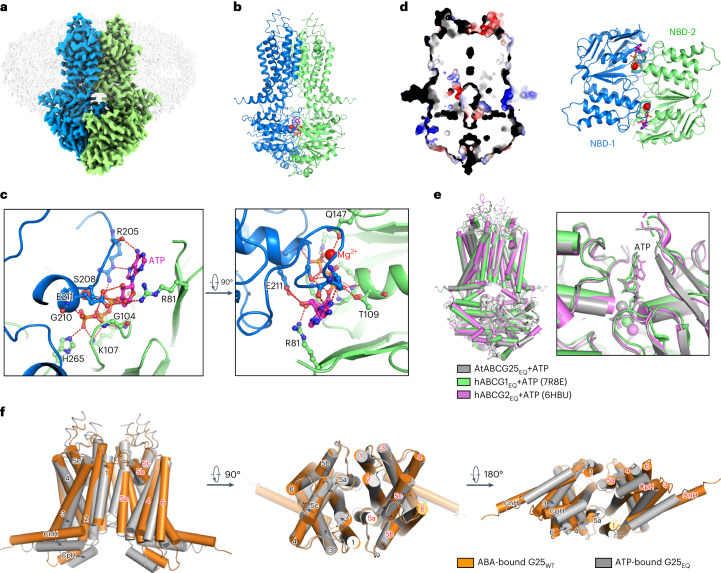
Architecture of ABCG25_EQ_ in the ATP-bound state. **a**, Overview of the electron density for the ABCG25 E232Q mutant in the ATP-bound state. Densities corresponding to the ABCG25 monomers are coloured blue and green. The detergent density is shown in grey. **b**, Overall structure of the ATP-bound ABCG25_EQ_ and the EM density for the ATP molecule (with nitrogen and phosphorus atoms coloured magenta and orange, respectively) and magnesium (coloured red). **c**, Coordination of ATP and Mg^2+^ by ABCG25. The ATP molecule and interacting residues are shown as sticks in the zoom-in views. Hydrogen bonds are shown as red dashed lines. **d**, Section view of the surface electrostatic potential of the ATP-bound ABCG25_EQ_ (left) and a cartoon representation of the NBDs with the ATP-binding site (right). **e**, Structure alignments of ABCG25_EQ_, hABCG1_EQ_ (PDB code: 7R8E) and hABCG2_EQ_ (PDB code: 6HBU) in the ATP-bound state. ABCG25, hABCG1 and hABCG2 are coloured grey, green and pink, respectively. **f**, Comparison of the TMDs of the ABA-bound ABCG25_WT_ (coloured orange) and the ATP-bound ABCG25_EQ_ (coloured grey) structures.

**Extended Data Fig. 8:**
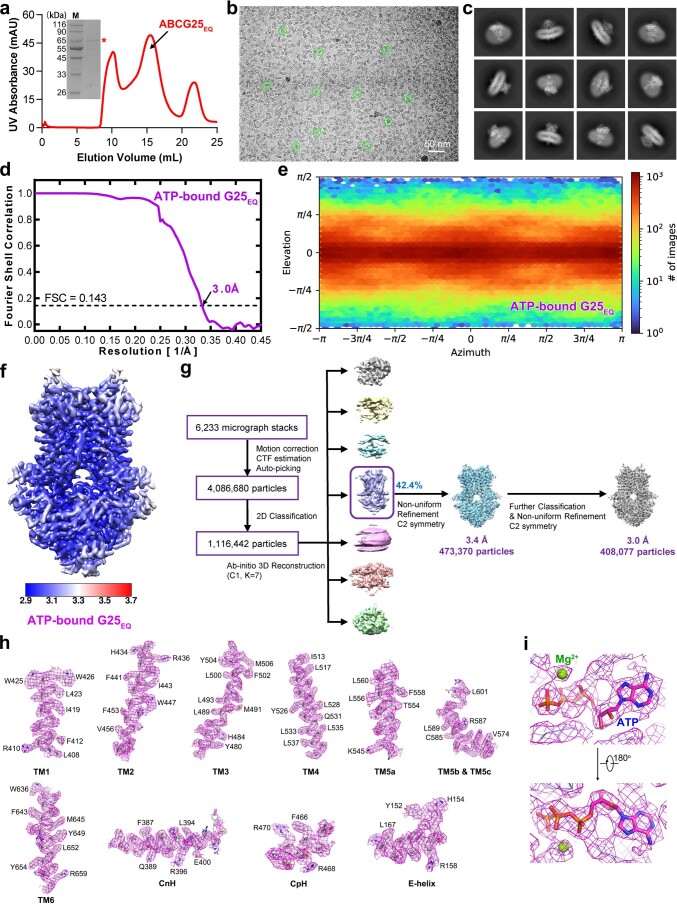
Cryo-EM analysis of ABCG25_EQ_ in the ATP-bound state. **a**, A representative gel filtration and Coomassie-blue-staining SDS-PAGE result of the E232Q mutant of ABCG25 (ABCG25_EQ_). Experiments have been repeated for at least three times with similar results. Uncropped gel can be found in Source Data. **b**, A typical cryo-EM image of the ATP-bound ABCG25_EQ_. Experiments have been repeated for at least three times with similar results. **c**, Typical 2D classification images of the ATP-bound ABCG25_EQ_. Experiments have been repeated for at least three times with similar results. **d**, The gold-standard Fourier shell correlation curve for the overall map of the ATP-bound ABCG25_EQ_. The resolution reaches 3.0 Å by a threshold of 0.143. e, Euler angle distribution of the ATP-bound ABCG25_EQ_. **f**, The local resolution map of the ATP-bound ABCG25_EQ_ calculated using ResMap. **g**, A flowchart for the data processing of the ATP-bound ABCG25_EQ_. **h**, The EM densities for the six transmembrane helices, CnH, CpH and E-helix of the ATP-bound ABCG25_EQ_ map. Residues with notable side chains are labeled aside. **i**, The EM densities of ATP and Mg^2+^ in the ATP-bound ABCG25_EQ_ map. Source data

ATP binding is supposed to induce conformational changes to ABC transporters and facilitate substrate translocation^50^. The ABCG25_EQ_ mutant had an almost abolished ATP hydrolysis activity (Fig. 1c), which is suitable to trap ABCG25 in the ATP-bound state. We purified this mutant and incubated it with ATP and Mg^2+^ before cryo-sample preparation (Extended Data Fig. 8a). An EM map with an overall resolution of 3.0 Å was finally obtained (Fig. 4a and Extended Data Fig. 8b–h). Clear densities were observed in each NBD, fitting well with the ATP molecule and Mg^2+^ (Fig. 4b and Extended Data Fig. 8i). ATP forms intense interactions with residues from both NBDs, thus pulling NBDs close together (Fig. 4b,c and Extended Data Fig. 7f). From one NBD, it is coordinated by G104, K107 and T109 of the Walker A motif, Q174 of the Q-loop, R81 and H265, by forming hydrogen bonds or salt bridges (Fig. 4c). From another NBD, it is coordinated by R205, S208, G210 and E211 of the signature sequence (Fig. 4c). A section view of the electrostatic potential surface reveals that the intracellular cavity observed in the apo or ABA-bound state disappears and the transmembrane region of ABCG25 is sealed to both sides of the membrane (Fig. 4d). Although from different species and with large variations in function and protein sequence (Extended Data Fig. 2), the ATP-bound state of *Arabidopsis* ABCG25 aligns better with the structures of hABCG1_EQ_ or hABCG2_EQ_ mutants in the same state (RMSDs of 1.97 Å and 1.83 Å, respectively) than with the alignment results of the apo-state structures (Fig. 4e). The TMDs from three proteins align well except for changes in TM5b and the extracellular loop between TM5c and TM6. The NBDs also align well with a quite similar ATP binding profile (Fig. 4e, left), and key residues involved in ATP-binding and hydrolysis are highly conserved among *Arabidopsis* ABCG25, hABCG1 and hABCG2 (Extended Data Fig. 2).

### Structure transitions between the ABA- and ATP-bound states

Structural alignments between the ABA-bound state of ABCG25_WT_ and the ATP-bound state of ABCG25_EQ_ reveal an RMSD of 6.29 Å (Extended Data Fig. 7g). As mentioned above, ATP binding leads to closure of the NBDs and such large movements are transduced to the TMDs through the three-helix bundle formed by CnH, CpH of the TMD and E-helix of the NBD. Compared with the ABA-bound state, all three helices are shifted by about one helix turn towards the opposing subunit (Extended Data Fig. 7h). The orchestrated movement of the three-helix bundle is facilitated by their intense interactions (Extended Data Fig. 7i,j). As observed in either the ABA-bound state or ATP-bound state structure, CnH is hydrophobically packed against the E-helix. In the ABA-bound state, a loop before the E-helix interacts with the CnH and CpH (Extended Data Fig. 7i). In the ATP-bound state, Y152 of this loop also forms a triad with R396 of CnH and E469 of CpH (Extended Data Fig. 7j). Notably, during structure transitions, the C-terminal part of CnH (R396 to H403) also undergoes straightening of the helix and rotates about 35°, which is not observed in other ABCG structures (Extended Data Fig. 7h).

As a result of the coupled movements of CnH, CpH and the E-helix, the two TMDs are largely rotated towards each other and close the gate at the inner leaflet of the membrane. This rotation is observed for all TMs from TM1 to TM6 in the intracellular part (Fig. 4f). The rotation is mostly rigid, as shown by structural alignments of the TMD from one subunit, except for TM1 (Extended Data Fig. 7k). F457, F453, V449 and I445 from opposing subunits stack together and form layers of gate on this side, hence may push the substrate to translocate along this path to the extracellular space (Extended Data Fig. 5d). However, in the determined ATP-bound ABCG25_EQ_ structure, the extracellular parts of the TMs remain mostly unshifted, except for slight shifts in TM2, TM5b and TM5c (Fig. 4f). The extracellular gate remains closed at this state, with Y564 and Y565 forming the plugging residues (Extended Data Fig. 5d). Thus, the ATP-bound structure of ABCG25_EQ_ may represent a state during the transport cycle when the bound ABA is released from the intracellular cavity when the extracellular gate is transiently open, and the extracellular gate closes again, ready to reset to the resting state after ATP hydrolysis and ADP release.

## Discussion

**Fig. 5:**
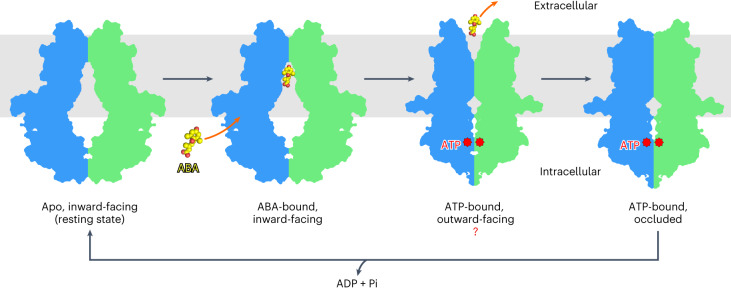
A proposed model for the ABCG25-mediated ABA efflux cycle. The ABA efflux cycle starts with the inward-facing (resting) state, in which ABCG25 is ready for ABA binding. Then, the ABA molecule binds to ABCG25 from the intracellular side. ATP binding to the NBDs of ABCG25 leads to closing of the NBDs and the conformational changes are transmitted to the TMDs through the three-helix bundles formed by CnH, CpH and E-helix. The inner parts of the TMs rotate to close the cavity and push the bound ABA to translocate along the transport path. The extracellular gate transiently opens and ABA is released to the extracellular side. ATP hydrolysis and release of ADP from NBDs reset ABCG25 to the resting state, ready for the next transport cycle. Source data.

**Extended Data Fig. 9:**
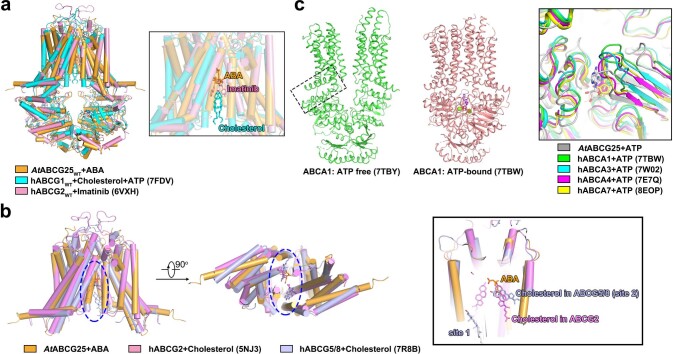
Structural comparisons between ABCG25 in Arabidopsis and human ABCs. **a**, Structure alignments of ABCG25_WT_ and hABCG1_WT_ (PDB code: 7FDV), hABCG2_WT_ (PDB code: 6VXH) in the substrate/inhibitor-bound state. **b**, Superposition of the ABA-bound ABCG25, the cholesterol-bound hABCG2 (PDB code: 5NJ3) and the cholesterol-bound hABCG5/8 (PDB code: 7R8B). **c**, Conserved ATP-induced conformation changes between ABCG25 and ABCAs. Shown here are the representative human cholesterol transporter ABCA1 in the ATP-free (PDB code: 7TBY) and ATP-bound (PDB code: 7TBW) state, respectively. The connecting helices are indicated by the dashed-line box. The large extracellular domain of ABCA1 is hidden. The inner panel shows structure alignments of the ATP binding site in ABCG25, hABCA1 (7TBW), hABCA3(7W0W), hABCA4 (7E7Q) and ABCA7 (8EOP) in the ATP-bound state.

As a mobile signalling molecule, the local and/or long-distance transport of ABA plays an essential role in its spatial distribution and physiological functions^25^. Since ABA is synthesized inside cells, the efflux of ABA to the extracellular space would be the first step of its movement, except when moving through plasmodesmata. As the first ABA exporter identified in plants, ABCG25 in *Arabidopsis* has been proposed to export ABA synthesized in vascular tissues to the guard cells, thus regulating stomatal closure^35^. In this study, with a newly set up ABA efflux assay system based on Sf9 insect cells and biochemical characterizations, we further verify that ABCG25 functions as an ABA exporter. Through structural analysis, the three-dimensional (3D) architectures of ABCG25 in the apo state, the ABA-bound state and the ATP-bound state are determined. In the apo structure of ABCG25, although no ABA was added during the whole process, we observed two clear densities in the cytosolic-facing cavity. These densities were interpreted as sterol molecules which might be extracted endogenously from the Sf9 cells. When added to the protein sample, ABA competed with the endogenous compounds and bound to the same binding sites in ABCG25. Interestingly, in humans, several ABCG family proteins function as cholesterol exporters, such as ABCG1 and ABCG5/G8 whose structures have been determined^41–43,51–53^. In hABCG2, although not a transport substrate, cholesterol was also observed in the determined structure with antibody fragments^39^. In hABCG1, cholesterol was captured binding to a position much closer to the cytosol^41^ (Extended Data Fig. 9a), and in hABCG5/ABCG8 dimer, two cholesterol binding sites have been observed^43^: one within the inner leaflet of the membrane, between the TMDs (site 1), and the other in the middle of the membrane (site 2). This site 2 is similar to the observed ABA binding site of ABCG25 and the cholesterol binding site of hABCG2 (Extended Data Fig. 9b), suggesting a conserved cholesterol binding profile in these ABCGs. Although with a typically much lower level in plants than in animals, cholesterol and other predominant sterols (such as β-sitosterol, campesterol and stigmasterol) are not negligible and play a wide spectrum of functions in plants^54,55^. Thus, an intriguing question arises whether the sterols serve as transport regulators or substrates of ABCG25 in *Arabidopsis*. The ABA binding site of ABCG25 lies in the middle of the transmembrane region. It consists of mainly hydrophobic and polar residues, a conserved feature observed in the human ABCG1, ABCG2 and ABCG5/G8 structures. However, the key forming residues are not highly conserved among the ABA transporters identified in *Arabidopsis*, even between ABCG25 and another ABA exporter ABCG31 (Extended Data Fig. 1a). How different ABCGs recognize ABA and mediate ABA flow in opposite directions remains to be further investigated. We also noted that for the density observed in the ABA-bound state with ABCG25 purified with DDM plus CHS, alternative modelling can be carried out with the ABA ring group pointing upwards in the same site, indicating the existence of other states during ABA transport. For hABCG2 and *Arabidopsis* ABCG25, which transport hydrophilic molecules, all substrates are captured to a similar position in the middle of the TMDs. The substrates may enter directly from the vestibule formed by the NBDs to this favourable binding site. Notably, at the entrance of the cavity in the ABCG25’s TMD, we noticed the presence of a positively charged lysine residue, K545 (Fig. 3d). The K545A mutant has a reduced ABA efflux activity compared with the ABCG25_WT_ (Fig. 3e), indicating that this residue may be involved in the recruitment of the negatively charged substrate. The ATP-bound structure of ABCG25 reveals dramatic changes in both the NBD and TMD domains. The ATP-binding-induced NBD closure is transduced to the TMD via the three-helix bundle between NBD and TMD. Similar ATP-binding mode and coupling mechanisms are shared in mammalian ABCGs, as well as ABCAs such as the human cholesterol/lipid transporters ABCA1, ABCA3, ABCA4 and ABCA7, the structures of which have been determined^56–63^ (Extended Data Fig. 9c). Combined with the apo-state and ABA-bound-state structures of ABCG25, and the wealth of structural and biochemical analysis of ABCGs from humans and yeast^50,64–66^, we propose a model of the ABCG25-mediated ABA efflux (Fig. 5). In the resting state, the NBDs of opposing subunits are mainly separated and the TMDs of ABCG25 form a cavity ready for substrate binding. ABA enters from the cytosolic side and its binding leads to subtle changes in either the TMD or NBD. ATP binding to the NBDs of ABCG25 leads to closing of the NBDs, and the rotational and translational changes of the NBDs are transmitted to the TMDs. The inner parts of the TMs of opposing subunits move to close the cavity and push the bound ABA to translocate along the channel mainly formed by TM2 and TM5. The extracellular gate transiently opens and ABA is released to the extracellular space. Then, ATP hydrolysis and release from the NBD reset ABCG25 to the resting state, ready for the next transport cycle. Such a mechanism has been well elucidated for ABC transporters from all kingdoms of life and is applied to ABCG25 in *Arabidopsis*. Notably, a back-to-back study also reveals similar architectures and structural transitions of the same transporter^67^. The determined ATP-bound structure may be a turnover, occluded state after ABA is released. An ATP-bound, outward-facing structure of ABCG25 awaits to be determined to reveal the detailed mechanism of ABA release.

In conclusion, the structural and functional analyses of ABCG25 in *A. thaliana* presented here provide more insights into the ABA transport and signalling pathway in plants, and set up a framework for further structure-based functional analysis of ABCG25-mediated ABA efflux.

## Methods

### Cloning and protein expression

The DNA sequence of full-length *A. thaliana* ABCG25 is publicly available at Uniprot (https://www.uniprot.org, accession code Q84TH5). The complementary DNA was cloned into the pFastBac vector (Invitrogen) with the N-terminal Flag tag (DYKDDDDK). Site-directed mutagenesis was performed using a standard two-step PCR and verified by DNA sequencing. Sequences of the oligonucleotides used in this study can be found in Supplementary Table 1. ABCG25_WT_ and its variants were expressed using the baculovirus system (Invitrogen). Briefly, bacmids were generated in DH10Bac cells (Invitrogen). The baculoviruses were generated and amplified in Sf9 cells (Invitrogen). Forty-eight hours after viral infection, cells were collected and resuspended in buffer containing 25 mM HEPES-NaOH (pH 7.4) and 150 mM NaCl. The suspension was supplemented with 1.5% (w/v) DDM (Anatrace) plus 0.3% (w/v) CHS (Sigma-Aldrich), or 1.5% (w/v) DDM alone and the protease inhibitor cocktail containing 1 mM phenylmethylsulfonyl fluoride, aprotinin (1.3 mg ml^−1^), pepstatin A (0.7 mg ml^−1^) and leupeptin (5 mg ml^−1^). After incubation at 4 °C for 2 h, the supernatant was isolated by centrifugation at 25,200 × *g* for 1 h and incubated with anti-Flag M2 affinity gel (Sigma) at 4 °C for 30 min.

For cryo-EM sample preparation, the resin was rinsed three times with buffer A containing 25 mM HEPES-NaOH (pH 7.4), 150 mM NaCl and 0.06% (w/v) digitonin (Apollo), and then eluted with buffer A plus 200 μg ml^−1^ Flag peptide. The protein eluent was concentrated using a 100-kDa cut-off Centricon (Millipore) and then applied to a Superose 6 column (GE Healthcare) in buffer B containing 25 mM HEPES-NaOH (pH 7.4), 150 mM NaCl and 0.03% (w/v) digitonin. Peak fractions were pooled together and further concentrated to ~11 mg ml^−1^ before cryo-EM sample preparation. Protein purification and sample processing were carried out at 4 °C.

For further ATPase activity assay and proteoliposome reconstitution, ABCG25_WT_ and the variants were expressed and purified in the same way, except that the detergent for buffer A was substituted with 0.02% (w/v) DDM and the detergent for buffer B was substituted with 0.01% (w/v) DDM. Peak fractions were pooled together and concentrated to ~3 mg ml^−1^ for subsequent assay.

### Cell-based ABA transport assay

Sf9 cells at a density of 2 × 10^6^ cells per ml were transfected with the virus of the WT or mutants of ABCG25. Forty-eight hours after transfection, cells were collected by centrifugation at 800 *g* for 5 min and resuspended for the loading assay or efflux assay. Cell counts were determined using Coulter counting and microscopic visualization. For all assay systems, a 500 μl aliquot of cell suspension contains 3 × 10^6^ cells. For the [^3^H]-ABA accumulation assay, cells were resuspended and incubated with phosphate-buffered saline (PBS) citrate buffer (pH 5.5, 10 mM Na_2_HPO_4_, 1.8 mM KH_2_PO_4_, 2.7 mM KCl, 137 mM NaCl, pH adjusted with anhydrous citric acid) containing 6.6 nM [^3^H]-ABA (specific activity 15 Ci mmol^−1^, American Radiolabeled Chemicals). The loading process was stopped by centrifugation at indicated timepoints. For the ABCG25 variants, the loading process was stopped at 10 min. Cells were then washed twice with ice-cold PBS citrate buffer (pH 5.5) and resuspended with the same buffer plus 1% Triton X-100 for cell lysis. Radioactivity was determined using a Tri-Carb 2910TR liquid scintillation counter (PerkinElmer). For the ABA efflux assay, cells were first loaded in PBS citrate buffer (pH 5.5) plus 6.6 nM [^3^H]-ABA for 10 min, then washed and resuspended with [^3^H]-ABA-free PBS citrate buffer. Aliquots (500 μl) were taken immediately after resuspension (defined as the zero timepoint) or at other indicated timepoints. Cells were centrifuged and washed twice with 1 ml ice-cold PBS citrate buffer (pH 5.5) and resuspended with the same buffer plus 1% Triton X-100 for scintillation counting.

### Proteoliposome reconstitution for ATPase assay

Proteoliposomes were prepared as previously described^68^. Briefly, *E. coli* polar lipid extract (Avanti):POPC (Avanti, 18:1–16:0 PC) at a 7:3 ratio (w/w) were dissolved in a mixture of methanol:chloroform (3:1 (v/v), Sigma-Aldrich) at 50 mg ml^−1^, then dried under nitrogen gas. Lipids were resuspended at 20 mg ml^−1^ in the reaction buffer (25 mM HEPES-NaOH, pH 7.4, 150 mM NaCl, 10 mM MgCl_2_), frozen and thawed 10 times in liquid nitrogen. For subsequent reconstitution, liposomes were extruded 31 times through 0.4 mm membrane filters (Cytiva) and incubated with 1% (w/v) DDM at 4 °C for 1 h. The detergent-solubilized protein was added into DDM-destabilized liposomes at a protein:lipid ratio of 1:100 (w/w) and incubated at 4 °C for 1 h. DDM was removed by adding 100 mg ml^−1^ biobeads (BioRad) in four batches overnight. The proteoliposomes were then homogenized in five freeze–thaw cycles and extruded again. Proteoliposomes were collected by ultracentrifugation at 160,000 × *g* for 1 h and resuspended in the reaction buffer (25 mM HEPES-NaOH, pH 7.4, 150 mM NaCl, 10 mM MgCl_2_) to a final lipid concentration of 100 mg ml^−1^.

### ATPase activity assay

ATPase activities of ABCG25_WT_ and its mutants in detergent micelle or liposomes were measured using the ATPase Colorimetric Assay kit (Innova Biosciences) in 96-well plates at an optical density of 650 nm. To calculate the ATPase activity in the presence of different concentrations of ATP, 1 μg protein was added to the reaction buffer containing 25 mM HEPES-NaOH (pH 7.4), 150 mM NaCl, 10 mM MgCl_2_, with (for ABCG25 in detergent) or without (for ABCG25 in liposomes) 0.01% (w/v) DDM. Then, ATP in different concentrations was supplemented in the solution to start the reaction at 37 °C for 10 min. The amount of released Pi was quantitatively measured with the ATPase Colorimetric Assay kit. For ABA concentration-dependent ATPase activity assay, a stock solution of ABA ((S)-(+)-Abscisic acid, Sigma) was prepared in double-distilled H_2_O at 20 mM concentration and then serially diluted to obtain the desired final concentrations. The ABA solution in different concentrations was added into the reaction buffer containing 1 μg protein and the solution mixed for a 20-min incubation on ice. Afterwards, ATP was supplemented into the mixture at a final concentration of 3 mM and reacted at 37 °C for 10 min. All statistical analyses were performed using GraphPad Prism 9.

### Cryo-EM sample preparation and data acquisition

For the apo-state ABCG25 sample, a 4 μl aliquot of purified ABCG25_WT_ protein was applied to a glow-discharged holey carbon grid (Quantifoil Cu R1.2/1.3, 300 mesh), blotted with a Vitrobot Mark IV (ThermoFisher) operating at 8 °C and 100% humidity using a blotting time of 3.0 s and plunged into liquid ethane cooled by liquid nitrogen. For the ABA-bound ABCG25 sample, a 4 μl aliquot of purified ABCG25_WT_ protein was incubated with 2 mM ABA on ice for 60 min before grid preparation. For the ATP-bound ABCG25 sample, a 4 μl aliquot of purified E232Q ABCG25 protein was incubated with 5 mM ATP and 10 mM MgCl_2_ on ice for 60 min before grid preparation.

The grids were loaded into a Titan Krios (FEI) electron microscope operating at 300 kV and equipped with the BioQuantum energy filter and K3 direct electron detector (Gatan). Images were recorded using EPU software in the super-resolution mode, with a calibrated pixel size of 0.55 Å at a nominal magnification of ×81,000 and defocus values from −1.5 to −2.3 μm. Each stack was acquired with an exposure time of 4 s and dose fractionated to 32 frames with a total dose of 50 e^−^ Å^−2^.

### Image processing

Flowcharts for the data processing is presented in Extended Data Figs. 4a,b and 8g. Motion correction and dose weighting were performed using the RELION 3.1 implementation of MotionCor2 (refs. ^69,70^) and the stacks were binned twofold, resulting in a pixel size of 1.1 Å. Subsequent data processing was all carried out with the twofold-binned micrographs. Meanwhile, defocus values were estimated using CTFFIND4 (ref. ^71^).

For the apo-state ABCG25 (with DDM plus CHS extraction), 549,613 particles were automatically picked from 2,147 micrographs using cryoSPARC (v.3.2.0)^72^. After several rounds of heterogeneous refinement, 268,502 particles were selected for ab initio reconstruction. After non-uniform refinement, an EM map at 3.1 Å was obtained with 208,162 particles. Further heterogeneous refinement, ab initio classifications and non-uniform refinement improved the resolution, and 174,479 particles were used for the final 3D reconstruction applying C2 symmetry at 3.0 Å. For the data processing of the ABA-bound ABCG25 (with DDM plus CHS extraction), similar procedures were carried out and an EM map with an overall resolution of 3.0 Å using 322,151 particles was obtained. For the data processing of the ATP-bound ABCG25, similar procedures were carried out and an EM map with an overall resolution of 3.0 Å using 408,077 particles was obtained. All resolutions were estimated with the gold-standard Fourier shell correlation at a 0.143 criterion with a high-resolution noise substitution method^73,74^. Local resolution variations were estimated using ResMap^75^. Similar data processing procedures were performed for the apo-state ABCG25 (with DDM extraction) and ABA-bound ABCG25 (with DDM extraction and ABA supplementation during all purification steps).

### Model building and refinement

The initial models were built by docking the predicted model by Alphafold2 of ABCG25 (AF-Q84TH5-F1) into the cryo-EM maps using UCSF Chimera^76,77^. Models were then adjusted and de novo built on the basis of the cryo-EM densities using Coot^78^. ABA, ATP and magnesium were from the ligand library in Coot. Structure refinements were carried out using PHENIX in real space^79^. Overfitting of the model was monitored by refining the model in one of the two independent maps from the gold-standard refinement approach and testing the refined model against the other map. Statistics of the 3D reconstruction and model refinement can be found in Extended Data Table 1.

### Western blotting analysis

The whole cell lysate samples expressing ABCG25_WT_ or mutants were run on BeyoGel Plus Precast PAGE gel (8–20%) for the Tris-Gly system (Beyotime) for 120 min at 100 V. The gel was transferred to a polyvinylidene fluoride membrane (Merck Millipore) with the Trans-Blot SD semi-dry electrophoretic transfer system (BioRad). The membrane was blocked with 5% (w/v) non-fat milk (Sangon Biotech) for 1 h at room temperature, followed by incubation with the primary antibody (anti-FLAG tag mouse monoclonal antibody, 1:3,000, CWBIO) for 1 h at room temperature. Then the membrane was washed 5 times with TBST buffer (20 mM Tris-HCl, pH 7.0, 150 mM NaCl, 0.1% (w/v) Tween-20) before incubation with the secondary antibody (HRP-conjugated goat-anti-mouse IgG, 1:5,000, CWBIO) for 1 h at room temperature. The membrane was washed 5 times and imaged after incubation with SuperSignal West Pico PLUS chemiluminescent substrate (Thermo Scientific).

### Immunofluorescence

Sf9 insect cells were grown on coverslips in 35 mm plates and transfected with the virus of the WT or mutants of ABCG25. Forty-eight hours after transfection, cells were fixed in 4% (w/v) paraformaldehyde for 30 min at 4 °C, subsequently permeabilized and blocked with PBS containing 0.3% (w/v) Triton X-100 and 3% (w/v) bovine serum albumin for 1 h at room temperature. For immunostaining, samples were incubated with the anti-Flag tag (DYKDDDDK) Alexa Fluor 594-conjugated antibody (1:50, CST) for 1 h at 37 °C, followed by incubation with 5 μM Dio (Absin) for 20 min at 37 °C. Nuclei were visualized with Hoechst 33342 (LEAGENE). All fluorescence images were captured via the LSM880 confocal laser scanning microscope (Zeiss) and analysed with ZEN (Zeiss).

### Reporting summary

Further information on research design is available in the Nature Portfolio Reporting Summary linked to this article.

### Supplementary information

Supplementary Information Supplementary Table 1.

Reporting Summary

### Source data

Source Data Figs. 1 and 3 and Extended Data Fig. 5 Statistical source data for Figs. 1a–f and 3e and Extended Data Fig. 5e–f.

Source Data Extended Data Figs. 3, 5, 6 and 8 Unprocessed gel for Extended Data Fig. 3a, unprocessed blot for Extended Data Fig. 5i and unprocessed gels for Extended Data Figs. 6e,h and 8a.

## Figures and Tables

**Extended Data Table 1:**
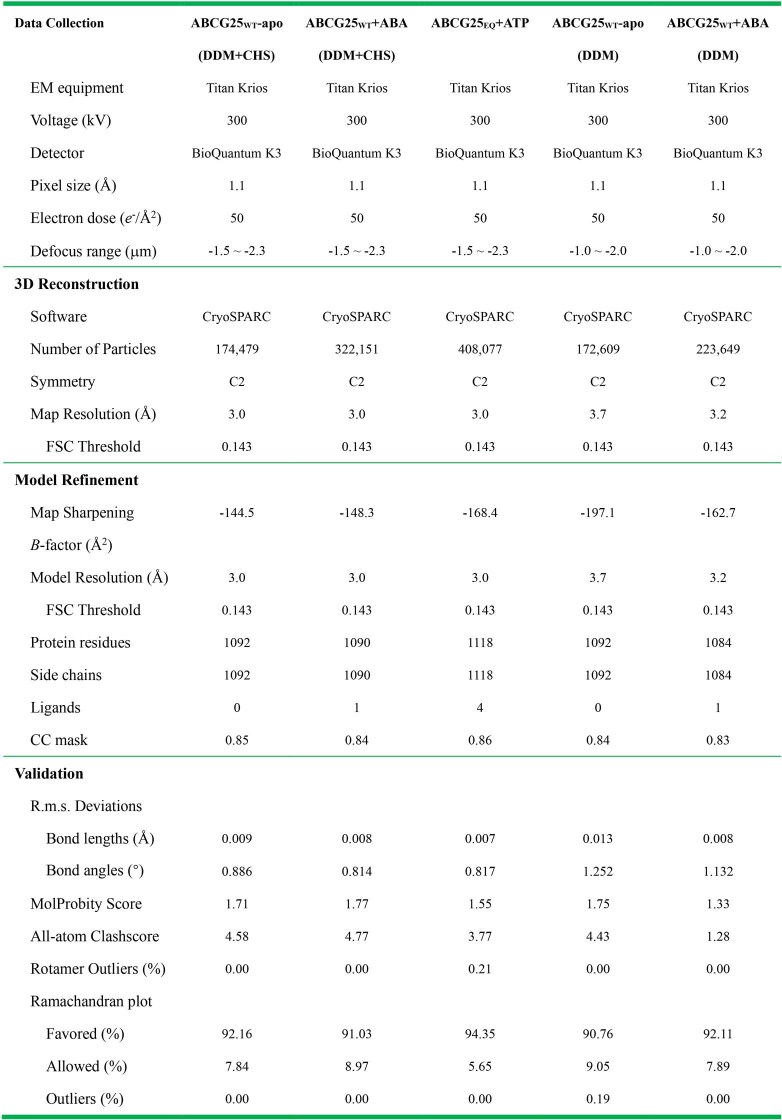
Statistics of cryo-EM data collection, processing, model refinement and validation

## Data Availability

The 3D cryo-EM density maps of the apo (DDM plus CHS extraction), apo (DDM extraction), ABA-bound (DDM plus CHS extraction), ABA-bound (DDM extraction with ABA added during whole purification steps) and ATP-bound dimeric state ABCG25 have been deposited in the Electron Microscopy Data Bank (EMDB, https://www.ebi.ac.uk/emdb/) under the accession numbers EMD-35768, EMD-36781, EMD-35769, EMD-36780 and EMD-35774, respectively. Coordinates for the reciprocal structures model have been deposited in the Protein Data Bank (PDB, https://www.rcsb.org/) under the accession codes 8IWJ, 8K0Z, 8IWK, 8K0X and 8IWN, respectively. Source data are provided with this paper.
