# Supplementary material for: Structural basis for abscisic acid efflux mediated by ABCG25 in Arabidopsis thaliana
Source: Nat Plants. 2023 Sep 4;9(10):1697–708. doi: 10.1038/s41477-023-01510-0 (PMC10581904; doi:10.1038/s41477-023-01510-0)
Supplement: Supplementary file 1 — Supplementary Table 1. [file 41477_2023_1510_MOESM1_ESM.pdf]

nature plants

Article

https://doi.org/10.1038/s41477-023-01510-0

Structural basis for abscisic acid efflux mediated by ABCG25 in *Arabidopsis thaliana*

In the format provided by the authors and unedited

Supplementary Table 1 | Primers used in this study.

Primer name	Primer sequence
**ABCG25-FW**	GATGACGATGACAAGCATATGTCAGCTTTTGACGGCGTTG
**ABCG25-RV**	GTACCGCATGCCTCGACCTCGAGTTAATGTTTGATACGTCTCAAAG
**E232Q-FW**	GAGTCTTTTGGTTCTTGATCAGCCTACTTCGGGTCTTGATG
**E232Q-RV**	CATCAAGACCCGAAGTAGGCTGATCAAGAACCAAAAGACTC
**Q413A-FW**	CTTCGCATTTTCGCAGTCGTCGCGGCTTCGATC
**Q413A-RV**	GAAGCCGCGACGACTGCGAAAATGCGAAGTAAGTC
**I445A-FW**	CTTCTTCATATCCGCCTTTTGGGGAGTACTTCCATC
**I445A-RV**	GTACTCCCCAAAAGGCGGATATGAAGAAGAGTAG
**V449A-FW**	CATCTTTTGGGGAGCACTTCCATCATTTAACGCGGTC
**V449A-RV**	GTTAAATGATGGAAGTGCTCCCCAAAAGATGGATATG
**F453A-FW**	GTACTTCCATCAGCTAACGCGGTCTTCACGTTTC
**F453A-RV**	GAAGACCGCGTTAGCTGATGGAAGTACTCCCCAAAAG
**T552A-FW**	GTCTACAATCGTGGCCGTGACAATGCTAGCGTTTG
**T552A-RV**	CTAGCATTGTCACGGCCACGATTGTAGAC
**L556A-FW**	GACCGTGACAATGGCAGCGTTTGTCTTAACCGGTG
**L556A-RV**	GTTAAGACAAACGCTGCCATTGTCACGGTCACGATTG
**V559A-FW**	CAATGCTAGCGTTTGCCTTAACCGGTGGTTACTAC
**V559A-RV**	GTAACCACCGGTTAAGGCAAACGCTAGCATTGTCAC
**Y565A-FW**	CTTAACCGGTGGTTACGCCGTCAACAAAGTGCCATC
**Y565A-RV**	CACTTTGTTGACGGCGTAACCACCGGTTAAGAC
**K545A-FW**	GATCATGGACGCTGCAAAAGCGTCTACAATCGTG
**K545A-RV**	GATTGTAGACGCTTTTGCAGCGTCCATGATCGCTGC
