# Supplementary material for: Structural basis for abscisic acid efflux mediated by ABCG25 in Arabidopsis thaliana
Source: Nat Plants. 2023 Sep 4;9(10):1697–708. doi: 10.1038/s41477-023-01510-0 (PMC10581904; doi:10.1038/s41477-023-01510-0)
Supplement: Supplementary file 2 — Reporting Summary [file 41477_2023_1510_MOESM2_ESM.pdf]

nature portfolio

Corresponding author(s): Xin Liu, Linfeng Sun

Last updated by author(s): Jul 21, 2023

Reporting Summary

Nature Portfolio wishes to improve the reproducibility of the work that we publish. This form provides structure for consistency and transparency in reporting. For further information on Nature Portfolio policies, see our Editorial Policies and the Editorial Policy Checklist.

Statistics

For all statistical analyses, confirm that the following items are present in the figure legend, table legend, main text, or Methods section.

n/a	Confirmed	
☐	☒	The exact sample size (*n*) for each experimental group/condition, given as a discrete number and unit of measurement
☐	☒	A statement on whether measurements were taken from distinct samples or whether the same sample was measured repeatedly
☐	☒	The statistical test(s) used AND whether they are one- or two-sided<br>*Only common tests should be described solely by name; describe more complex techniques in the Methods section.*
☒	☐	A description of all covariates tested
☐	☒	A description of any assumptions or corrections, such as tests of normality and adjustment for multiple comparisons
☐	☒	A full description of the statistical parameters including central tendency (e.g. means) or other basic estimates (e.g. regression coefficient) AND variation (e.g. standard deviation) or associated estimates of uncertainty (e.g. confidence intervals)
☐	☒	For null hypothesis testing, the test statistic (e.g. *F*, *t*, *r*) with confidence intervals, effect sizes, degrees of freedom and *P* value noted<br>*Give P values as exact values whenever suitable.*
☒	☐	For Bayesian analysis, information on the choice of priors and Markov chain Monte Carlo settings
☒	☐	For hierarchical and complex designs, identification of the appropriate level for tests and full reporting of outcomes
☒	☐	Estimates of effect sizes (e.g. Cohen's *d*, Pearson's *r*), indicating how they were calculated

Our web collection on statistics for biologists contains articles on many of the points above.

Software and code

Policy information about availability of computer code

Data collection	EPU-2.12 for EM data collection, ZEN 2012 v.1.1.2.0 for immunofluorescence imaging
Data analysis	RELION 3.1, MotionCor2-1.3.2, CTFFIND4-4.1, cryoSPARC v.3.2.0, ResMap-1.1.5, COOT-0.8.6, PHENIX-1.18rc1-3777, GraphPad Prism 9, ZEN 2012 v.1.1.2.0, UCSF Chimera-1.15, PyMOL-2.3.3

For manuscripts utilizing custom algorithms or software that are central to the research but not yet described in published literature, software must be made available to editors and reviewers. We strongly encourage code deposition in a community repository (e.g. GitHub). See the Nature Portfolio guidelines for submitting code & software for further information.

Data

Policy information about availability of data

All manuscripts must include a data availability statement. This statement should provide the following information, where applicable:

- Accession codes, unique identifiers, or web links for publicly available datasets
- A description of any restrictions on data availability
- For clinical datasets or third party data, please ensure that the statement adheres to our policy

The 3D cryo-EM density maps of the apo (DDM plus CHS extraction), apo (DDM extraction), ABA-bound (DDM plus CHS extraction), ABA-bound (DDM extraction with ABA added during whole purification steps) and ATP-bound dimeric state ABCG25 have been deposited in the Electron Microscopy Data Bank (EMDB, https://www.ebi.ac.uk/emdb/) under the accession number EMD-35768, EMD-36781, EMD-35769, EMD-36780, and EMD-35774, respectively. Coordinates for the

reciprocal structures model have been deposited in the Protein Data Bank (PDB, https://www.rcsb.org/) under the accession code 8IWJ, 8K0Z, 8IWK, 8K0X, and 8IWN, respectively. Source data are provided with this paper.
Coordinates for the hABCG1, hABCG2, hABCG5/8, hABCA1, hABCA3, hABCA4, hABCA7 are publicly available at the PDB (https://www.rcsb.org/) with the following accession codes: hABCG2wt: 5NJ3, hABCG2wt+Mitoxantrone: 7NFD, hABCG1eq+ATP: 7R8E, hABCG2eq+ATP: 6HBU, hABCG1wt: 7R8C, hABCG1wt+cholesterol+ATP: 7FDV, hABCG2wt+Imatinib: 6VXH, hABCG5/8+cholesterol: 7R8B, hABCA1 ATP-free: 7TBY, hABCA1+ATP: 7TBW, hABCA3+ATP: 7W02, hABCA4+ATP: 7E7Q, hABCA7 +ATP: 8EOP.
Protein sequences for the ABCGs in Arabidopsis and human are publicly available at Uniprot (https://www.uniprot.org) with the following accession codes: AtABCG25: Q84TH5, AtABCG17: Q9M2V6, AtABCG18: Q9M2V5, AtABCG30: Q8GZ52, AtABCG31: Q7PC88, AtABCG40: Q9M9E1, hABCG1: P45844, hABCG2: Q9UNQ0, hABCG5: Q9H222, hABCG8: Q9H221.

Human research participants

Policy information about studies involving human research participants and Sex and Gender in Research.

Reporting on sex and gender	N/A
Population characteristics	N/A
Recruitment	N/A
Ethics oversight	N/A

Note that full information on the approval of the study protocol must also be provided in the manuscript.

Field-specific reporting

Please select the one below that is the best fit for your research. If you are not sure, read the appropriate sections before making your selection.

☒ Life sciences ☐ Behavioural & social sciences ☐ Ecological, evolutionary & environmental sciences

For a reference copy of the document with all sections, see nature.com/documents/nr-reporting-summary-flat.pdf

Life sciences study design

All studies must disclose on these points even when the disclosure is negative.

Sample size	All of functional assays were performed with at least three replicates and decribed in the figure legends. The sample size were chosen to ensure the reproducibility of the experiments and to get meaningful results. The sample size were adequate based on distribution of data points and clearly visible effects.
Data exclusions	No data were excluded from the analyses.
Replication	All of functional assays were repeated independently at least three times and all attempts at replication were successful. The number of replications are described in the text.
Randomization	For cryo-EM 3D refinement, all particles were randomly split into two groups. Samples were randomly extracted from the stock and prepared for the functional assays. But for the following steps of all biochemical assays, randomization is not relevant to get solid and repeatable results.
Blinding	Blinding was not used in this study, because it is not technically or practically feasible to do so for either the cryo-EM structure determination or the functional assays. Blinding could not be used for the cryo-EM experiment as the protein sequence must be known for structure modelling. Blinding was also not relevant as all data were collected and processed in an automated fashion and fit when necessary by automated programs. For biochemical assays, the protein constructs need to be known before carry out the functional assays and for the subsequent comparisons.

Reporting for specific materials, systems and methods

We require information from authors about some types of materials, experimental systems and methods used in many studies. Here, indicate whether each material, system or method listed is relevant to your study. If you are not sure if a list item applies to your research, read the appropriate section before selecting a response.

Materials & experimental systems

n/a	Involved in the study	
☐	☒	Antibodies
☐	☒	Eukaryotic cell lines
☒	☐	Palaeontology and archaeology
☒	☐	Animals and other organisms
☒	☐	Clinical data
☒	☐	Dual use research of concern

Methods

n/a	Involved in the study	
☒	☐	ChIP-seq
☒	☐	Flow cytometry
☒	☐	MRI-based neuroimaging

Antibodies

Antibodies used

The anti-Flag antibody for western blotting was purchased from CoWin Biosciences (1:3000, Catalog number: CW0287, Lot number: 01222/12422). The HRP-conjugated goat-anti-mouse IgG was also purchased from CoWin Biosciences (1:5000, Catalog number: CW0102S, Lot number: 01325/33621). The anti-flag Alexa Fluor 594-conjugated antibody for immunofluorescence was purchased from CST (1:50, Catalog number: 20861S, Lot number: 2).

Validation

The anti-Flag antibody and HRP-conjugated goat-anti-mouse IgG for western blotting were validated by the commercial vendors:
anti-Flag: https://cwbio.com/goods/index/id/10178
HRP-conjugated goat-anti-mouse IgG: https://cwbio.com/goods/index/id/10118
The anti-flag Alexa Fluor 594-conjugated antibody for immunofluorescence was validated by the commercial vendors: https://www.cellsignal.com/products/antibody-conjugates/dykddddk-tag-d6w5b-rabbit-mab-binds-to-same-epitope-as-sigma-s-anti-flag-m2-antibody-alexa-fluor-594-conjugate/20861?_=1685788382551&Ntt=20861S&tahead=true

Eukaryotic cell lines

Policy information about cell lines and Sex and Gender in Research

Cell line source(s)

Sf9 cells (Invitrogen)

Authentication

No further authentication was performed for commercially available cell lines.

Mycoplasma contamination

The cell line has been tested negative for mycoplasma contamination.

Commonly misidentified lines
(See ICLAC register)

No commonly misidentified cell lines were used in this study.
