# Supplementary material for: Structural basis for abscisic acid efflux mediated by ABCG25 in Arabidopsis thaliana
Source: Nat Plants. 2023 Sep 4;9(10):1697–708. doi: 10.1038/s41477-023-01510-0 (PMC10581904; doi:10.1038/s41477-023-01510-0)
Supplement: Supplementary file 4 — Unprocessed gel for Extended Data Fig. 3a, unprocessed blot for Extended Data Fig. 5i and unprocessed gels for Extended Data Figs. 6e,h and 8a. [file 41477_2023_1510_MOESM4_ESM.pdf]

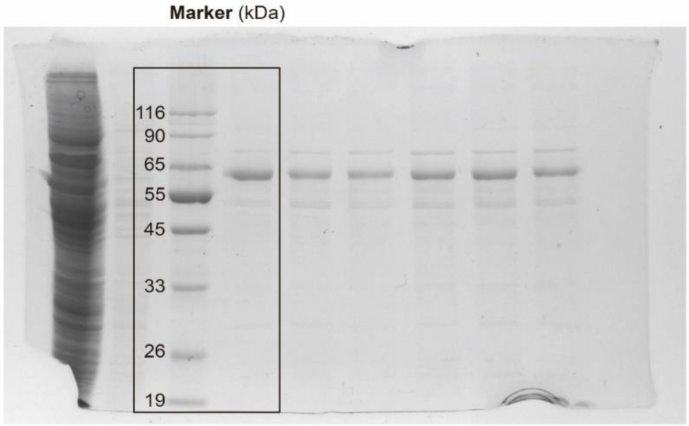

Uncropped SDS-PAGE gel for the WT *At*ABCG25 purified with DDM plus CHS extraction. Related to Extended Data Fig. 3a.

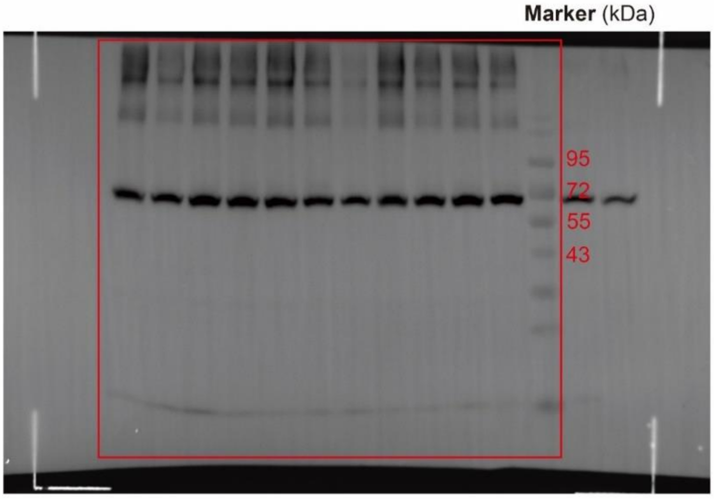

Uncropped Western blot of *At*ABCG25 variants using anti-Flag antibody. Related to Extended Data Fig. 5i.

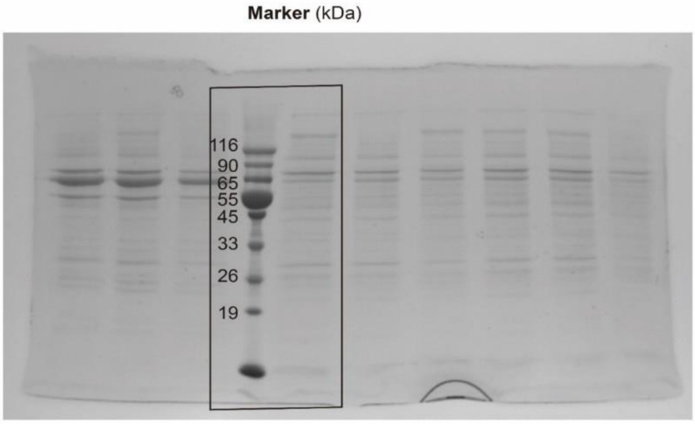

Uncropped SDS-PAGE gel for the WT *At*ABCG25 purified with DDM extraction. Related to Extended Data Fig. 6e.

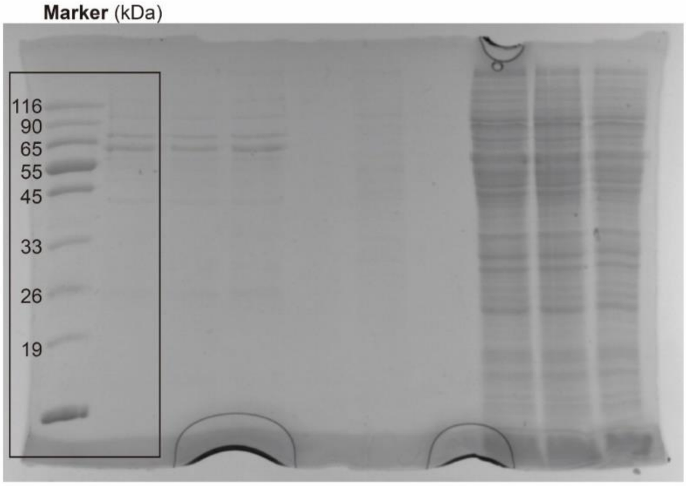

Uncropped SDS-PAGE gel for the WT *At*ABCG25 purified with DDM extraction and ABA-added during the whole purification steps. Related to Extended Data Fig. 6h.

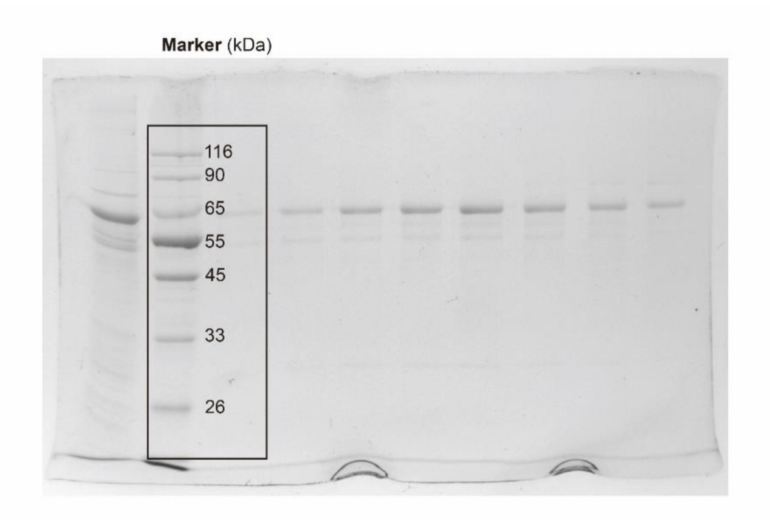

Uncropped SDS-PAGE gel for the E232Q mutant of *At*ABCG25. Related to Extended Data Fig. 8a.
